# BRAF inhibitors enhance erythropoiesis and treat anemia through paradoxical activation of MAPK signaling

**DOI:** 10.1038/s41392-024-02033-6

**Published:** 2024-12-02

**Authors:** Shunkang Wu, Yuelin Deng, Haobo Sun, Xuewen Liu, Shuo Zhou, Hanxi Zhao, Huan Li, Fusheng Guo, Qiuyu Yue, Fan Wu, Xinying Zhao, Na Li, Shicong Zhu, Qi Hu, Si Xie, Jie Zheng, Meng Lv, Yuan Kong, Xiao-Jun Huang, Xiaoguang Lei, Xiangmin Tong, Xiaofei Gao, Hsiang-Ying Lee

**Affiliations:** 1 The MOE Key Laboratory of Cell Proliferation and Differentiation, School of Life Sciences, Peking University, Beijing, 100871 China; 2 Peking-Tsinghua Center for Life Sciences, Academy for Advanced Interdisciplinary Studies, Peking University, Beijing, 100871 China; 3 School of Life Sciences, Westlake University, Hangzhou, Zhejiang China; 4 Westlake Laboratory of Life Sciences and Biomedicine, Hangzhou, Zhejiang China; 5 Department of Chemical Biology, College of Chemistry and Molecular Engineering, Peking University, Beijing, 100871 China; 6 Hematology Center, Beijing Key Laboratory of Pediatric Hematology Oncology; National Key Discipline of Pediatrics (Capital Medical University); Key Laboratory of Major Diseases in Children, Ministry of Education; Beijing Children’s Hospital, Capital Medical University, National Center for Children’s Health, Beijing, China; 7 Peking University People’s Hospital, Peking University Institute of Hematology, National Clinical Research Center for Hematologic Disease, Beijing Key Laboratory of Hematopoietic Stem Cell Transplantation, Collaborative Innovation Center of Hematology, Peking University, Beijing, 100871 China; 8 Department of Hematology, Zhejiang Provincial People’s Hospital, Hangzhou, Zhejiang China

**Keywords:** Drug screening, Molecular medicine

## Abstract

Erythropoiesis is a crucial process in hematopoiesis, yet it remains highly susceptible to disruption by various diseases, which significantly contribute to the global challenges of anemia and blood shortages. Current treatments like erythropoietin (EPO) or glucocorticoids often fall short, especially for hereditary anemias such as Diamond-Blackfan anemia (DBA). To uncover new erythropoiesis-stimulating agents, we devised a screening system using primary human hematopoietic stem and progenitor cells (HSPCs). We discovered that BRAF inhibitors (BRAFi), commonly used to treat BRAF^V600E^ melanoma, can unexpectedly and effectively promote progenitor cell proliferation by temporarily delaying erythroid differentiation. Notably, these inhibitors exhibited pronounced efficacy even under cytokine-restricted conditions and in patient samples of DBA. Mechanistically, although these BRAFi inhibit the MAPK cascade in BRAF^V600E^ mutant cells, they paradoxically act as amplifiers in wild-type BRAF cells, potently enhancing the cascade. Furthermore, we found that while the oncogenic BRAF^V600E^ mutation disrupts hematopoiesis and erythropoiesis through AP-1 hyperactivation, BRAFi minimally impact HSPC self-renewal and differentiation. In vivo studies have shown that BRAFi can enhance human hematopoiesis and erythropoiesis in severe immunodeficient mouse models and alleviate anemia in the *Rpl11* haploinsufficiency DBA model, as well as other relevant anemia models. This discovery underscores the role of the MAPK pathway in hematopoiesis and positions BRAFi as a promising therapeutic option for improving hematopoietic reconstitution and treating anemias, including DBA.

## Introduction

Erythropoiesis, essential for the daily production of roughly 2 × 10^11^ red blood cells, is a key process compromised in various hematologic disorders that lead to anemia.^1,2^ The supply and function of erythroid progenitors, notably burst-forming unit-erythroid cells (BFU-Es) and colony-forming unit-erythroid cells (CFU-Es), are critical for erythroid production.^3^ Erythropoietin (EPO) supports CFU-Es survival and differentiation and is a common treatment for anemia, especially in chronic kidney diseases.^4,5^ However, its effectiveness is limited in cases where progenitor cells are deficient, including anemias caused by hemolysis, sepsis, or genetic bone marrow failure diseases like Diamond-Blackfan anemia (DBA).^4,6–8^ Targeting BFU-E cells to enhance their self-renewal has emerged as a promising approach for treating EPO-resistant anemias. Agents like corticosteroids,^9^ HIF-PHD inhibitors,^10^ TGF-β inhibitors,^5^ and PPARα agonists^4^ can regulate BFU-Es proliferation but have not resolved all types of anemia. Further understanding of the molecular mechanisms governing BFU-E self-renewal remains critical for developing effective treatments for EPO-resistant anemias. The current limited range of pharmacological options underscores the pressing need to identify new therapeutic targets.^6^

The extracellular-signal-regulated kinase (ERK)/mitogen-activated protein kinases (MAPK) pathway is integral to mammalian cell proliferation, survival, and differentiation, significantly impacting erythropoiesis.^11,12^ Activation of this pathway is mediated by cytokines like EPO and stem cell factor (SCF), which bind to their respective receptors (EPOR and KIT), initiating downstream signaling processes.^13^ The SCF-KIT interaction is pivotal for the proliferation of erythroid progenitors, while EPO-EPOR signaling is crucial for the survival and maturation of late progenitors and erythroblasts in later stages. SCF and EPO synergistically activate key downstream signaling pathways, orchestrating erythroid development.^13,14^ The transition from self-renewal to differentiation in erythropoiesis necessitates a decline in KIT-mediated ERK/MAPK signaling, which coincides with the cell’s reduced proliferative capacity, as supported by recent proteomic and phospho-signaling studies.^15,16^

Mutations in the ERK/MAPK pathway occur in approximately 30% of cancers, with around 7% exhibiting mutations in the BRAF gene.^17^ The predominant BRAF mutation, BRAF^V600E^, found in over 90% of these cases, leads to constitutive activation of the ERK/MAPK signaling pathway.^18^ This is observed in multiple cancer types, including melanoma, thyroid cancer, and colorectal cancers.^17^ In hematopoietic stem and progenitor cells (HSPCs), the BRAF^V600E^ mutation disrupts normal hematopoiesis and erythropoiesis, contributing to conditions like hairy cell leukemia (HCL) and Langerhans cell histiocytosis (LCH).^19,20^

Here, we found that BRAF inhibitors (BRAFi), typically used to treat melanomas with the BRAF^V600E^ mutation, can prominently promote cell proliferation and erythropoiesis through amplifying MAPK activation via dimerizing with CRAF in wild-type BRAF cells. Demonstrating robust effects in vitro, these inhibitors also enhanced hematopoiesis and erythropoiesis in immunodeficient NPSG and NCG-X mice and various anemia models,^21^ including the *Rpl11* haploinsufficiency mouse model of DBA. Our research highlights BRAFi as a potential therapeutic candidate to enhance recovery from anemias or to promote ex vivo erythroid production.

## Results

### BRAF inhibitors promoted erythroid progenitor self-renewal in vitro

Targeting early erythroid progenitors, particularly BFU-Es, presents a viable therapeutic strategy for enhancing erythroid production, given their susceptibility to cell cycle modulation by small molecules such as glucocorticoids.^9^ Despite existing methodologies to purify BFU-Es, their scant presence presents significant hurdles for efficient compound screening.^22,23^ To address the challenges, we developed a 7-day screening protocol for erythroid differentiation and proliferation, utilizing primary human CD34^+^ HSPCs derived from cord blood (UCB-CD34^+^). This screening strategy effectively directed CD34^+^ cells through normal erythroid differentiation to become erythroid progenitors and precursors (CD71^+^CD235a^−^ and CD71^+^CD235a^+^) (Supplementary Fig. 1a, b). This streamlined process only requires a single administration of screening chemicals at the beginning, with cell number and viability readily evaluated using the CellTiter assay on Day 7.

We conducted a preliminary screening using an FDA-approved drug library to identify small molecule drugs that could enhance the expansion of erythroid progenitor cells. Our findings unexpectedly identified BRAF inhibitors, including Dabrafenib, Vemurafenib, and Encorafenib (Enco), as top candidates among FDA-approved agents for potentially promoting human erythroid cell proliferation (Supplementary Fig. 1c). Further exploration of additional BRAF inhibitors revealed GDC-0879 (GDC), SB-590885 (SB), and FDA-approved Encorafenib are significantly more potent in promoting the expansion of these progenitor cells (Supplementary Fig. 1c). Treatment with GDC and SB in the screening system not only enhanced the proliferation of progenitor cells but also raised their relative abundance (Supplementary Fig. 1d). Collectively, these findings indicate that BRAFi have the potential to stimulate erythropoiesis.

**Fig. 1:**
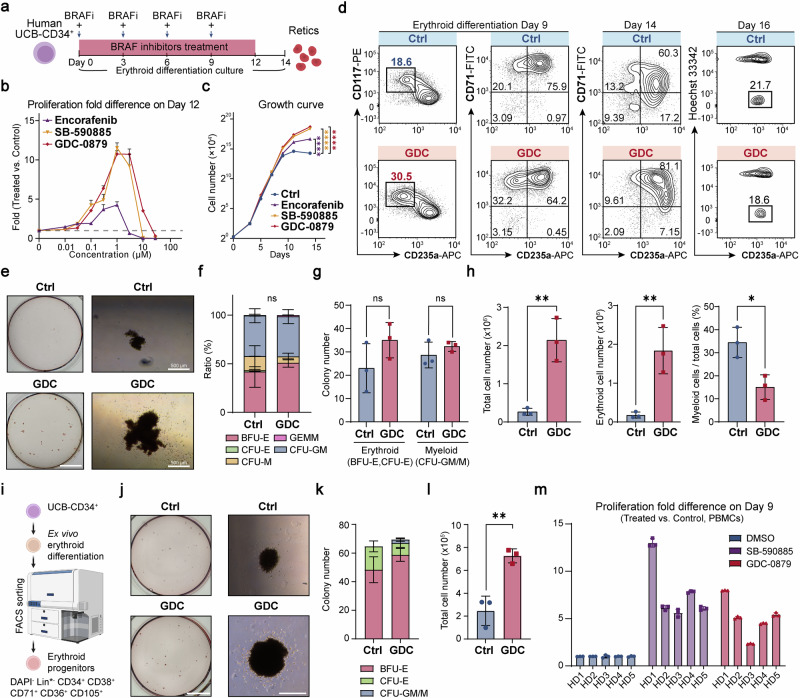
BRAF inhibitors promoted the self-renewal of primary erythroid progenitors in vitro**. a** Drug treatment workflow in UCB-CD34^+^-derived in vitro erythroid differentiation culture system. “+” indicates BRAFi treatment in conjunction with a change of medium. **b** The drug dose-response assay for UCB-CD34^+^-derived erythroid culture was conducted, with total cell numbers counted on Day 12. The graph illustrates the fold difference in proliferation between the GDC-treated and control (DMSO) groups on Day 12. The dashed line indicates the fold change for the control group. **c** The growth curve of UCB-CD34^+^-derived erythroid culture over 14 days in vitro, starting from 1.2 × 10^4^ cells on Day 0. The asterisks represent statistical differences obtained through two-way ANOVA test in cell number between the treatment groups and the control (DMSO) group. **d** Representative flow cytometry analysis of UCB-CD34^+^-derived erythroid cells in the control group (DMSO) and the GDC-0879-treated group on differentiation Day 9 (Left), Day 14 (Middle) and Day 16 (treated from Day 0–9) (Right). CD117 (c-kit), receptor for stem cell factor; CD71, transferrin receptor; CD235a (Glycophorin A), erythroid marker. CD235a^+^Hoechst^-^ cells are regarded as enucleated reticulocytes. **e** Representative images of colony forming assay (CFA) of 300 UCB-hCD34^+^ cells seeded in Methocult H4435 and cultured for 14 days. (Left) Whole-plate view; (Right) BFU-E colony. Scale bar = 10 mm (Left), 500 μm (Right). **f**, **g** Colony number and ratio statics of panel (**e**). There was no significant difference between the two groups in the proportion of either colony in panel (**f**). **h** Quantification of total cell numbers, erythroid cell numbers and the myeloid lineage ratio of cells washed from Methocult medium on Day 14 in the CFA of panel (**e**). Erythroid cells are identified as CD235a^+^, and myeloid cells as CD11b^+^. **i** UCB-CD34^+^ cells were differentiated for 5 days, after which erythroid progenitor cells were sorted and seeded in Methocult medium. (Bottom) Strategy for sorting erythroid progenitor cells. Lin* includes CD2, CD3, CD14, CD16, CD19, CD56, CD235a, CD45RA, CD123, CD7, CD10, CD90, CD135, and CD41a. **j** Representative images of colony forming assay of 100 erythroid progenitor cells seeded in Methocult H4435 and cultured for 14 days. (Left) Whole-plate view; (Right) BFU-E colony. Scale bar = 10 mm (Left), 500 μm (Right). **k**, **l** Colony number and quantification of cell number of cells washed from Methocult medium on Day 14 in the CFA of panel (**j**). **m** Fold change in cell number of PBMCs from 5 healthy donors (HDs) cultured in an erythroid differentiation system on Day 9. Unless otherwise noted, all experiments used control (DMSO), SB-590885 at 1 μM, GDC-0879 at 2 μM, and Encorafenib at 0.5 μM. BFU-E burst forming unit-erythroid, CFU-E colony forming unit-erythroid, CFU-M colony forming unit-monocyte, CFU-GM colony forming unit-granulocyte macrophage, GEMM Granulocytic-erythrocytic-megakaryocytic-macrophage. Error bars represent the mean ± SD from three biological replicates. A two-tailed unpaired Student’s *t*-test was performed for the statistical comparison between two groups (ns, *P* *>* 0.05; *P < 0.05; **P < 0.01; ***P < 0.001; ****P < 0.0001)

Regarding proliferation, dose-response assays in UCB-CD34^+^ erythroid differentiation cultures showed that GDC and SB induced a more than 10-fold increase in cell numbers at their optimal concentrations by Day 12, whereas Enco led to approximately a 4-fold increase compared to the control (Fig. 1a, b). By Day 14, GDC and SB had led to total cell number expansion by 354,567- and 281,516-fold, respectively, and Enco had resulted in an 86,381-fold expansion while control group only expanded by 16,573-fold from the start of the culture (Fig. 1c). Moreover, continued treatment with BRAFi caused a temporary delay in erythroid differentiation in the human CD34^+^ erythroid culture system between Day 5 and 14 (Fig. 1d, Supplementary Fig. 1e). Notwithstanding the initial delay, removing BRAFi in the latter stages (after Day 9) did not interfere with terminal differentiation, as indicated by the enucleation of erythroid cells (Fig. 1d, Supplementary Fig. 1f).

Subsequently, we investigated the impact of BRAFi on the lineage fate decisions of HSPCs. In the colony-forming assay, while BRAFi treatment modestly enhanced the total colony count and showed minimal effects on colony ratios or lineage determination, it notably augmented the size of BFU-E erythroid colonies (Fig. 1e, f). Moreover, treatment with BRAFi significantly increased overall cell numbers by nearly 10-fold, along with a substantial rise in erythroid cell numbers and a decrease of the proportion of other myeloid cells among those recovered from total colonies (Fig. 1e–h). To further examine the effect of BRAF inhibitors on erythroid progenitor cell proliferation, we sorted out erythroid progenitor cells and treated them with BRAF inhibitors.^24^ We observed that the BRAF inhibitor-treated group showed an increase in BFU-E colonies and a decrease in CFU-E colonies, with the total number of cells—primarily erythroid—recovered from the colonies increasing by approximately threefold in the BRAFi-treated group (Fig. 1i–l, Supplementary Fig. 1g). In addition to demonstrating the proliferative potential of BRAFi in early erythroid progenitor cells, we confirmed that BRAFi continued to exert a significant proliferative effect in later stages (erythroid differentiation Day 5-9 and Day 9-13) when administered for 4 days (Supplementary Fig. 1h). These findings suggest that BRAFi not only promotes the proliferation of early erythroid progenitor cells, such as BFU-Es, but also directly enhances the proliferation of precursor cells. Overall, the proliferative effect of BRAFi on erythroid cells is substantial and proportional to the duration of treatment.

Studies have shown that erythroid progenitors and precursors derived from human peripheral blood mononuclear cells (PBMCs) can be efficiently expanded and thus can serve as a valuable source for ex vivo RBC generation.^25^ Evaluations of PBMCs from healthy donors revealed that BRAFi markedly promoted erythroid proliferation, leading to a dramatic expansion of BFU-Es areas (Fig. 1m, Supplementary Fig. 1i–k), underscoring the impact of stimulating erythroid progenitors in enhancing PBMCs-derived erythropoiesis. Further experimentation demonstrated that BRAFi, particularly in combination with TGF-β inhibitor and glucocorticoids, synergistically increased the size of erythroid colonies, presenting a potent strategy to further enhance erythropoiesis (Supplementary Fig. 1l–n).

Taken together, these results demonstrate that BRAFi can promote erythroid progenitor self-renewal, leading to augmented erythroid output.

### BRAF inhibitors mitigate impaired erythropoiesis under cytokine-restricted conditions

**Fig. 2:**
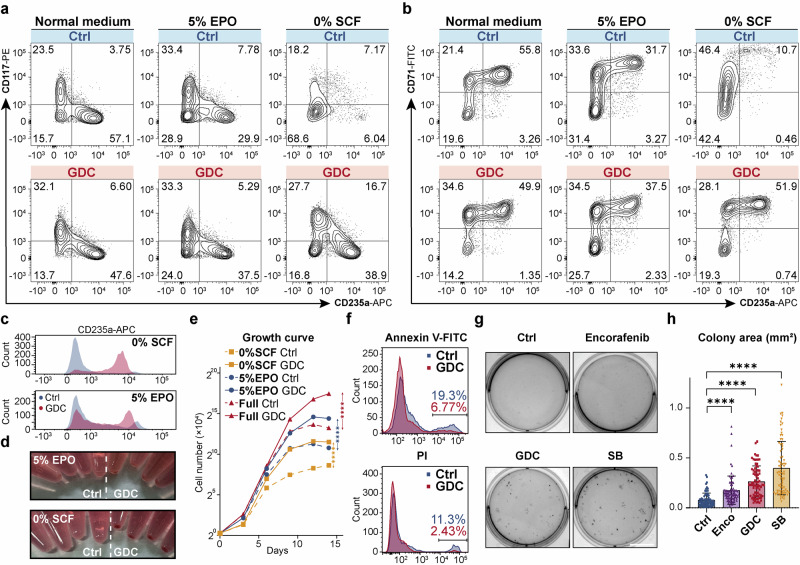
BRAF inhibitors attenuated ineffective erythropoiesis under cytokine-restricted conditions**. a**, **b** Flow cytometry analysis of UCB-CD34^+^-derived erythroid cells in the control group and GDC-0879-treated group on differentiation Day 9 under normal and specified conditions (5% EPO [0.15 IU/mL EPO]; 0% SCF [0 ng/mL SCF]), using cell surface markers CD117, CD235a, and CD71, CD235a. Concentrations of other cytokines were kept unchanged as usual. **c** Histograms showing the differences in CD235a levels between GDC-treated and control erythroid cells under different cytokine-restricted conditions on Day 14. **d** Cell pellets of UCB-CD34^+^ derived erythroid cells cultured under different cytokine-restricted conditions on Day 14, with each group containing equivalent cell numbers in this image. **e** Growth curves of UCB-CD34^+^-derived erythroid cells, starting from 1.2 × 10^4^ cells, treated with 2 μM GDC-0879 under the indicated conditions from Day 0 to Day 14. The asterisks indicate statistical differences in cell number between the treatment groups and the control group, as determined by a two-way ANOVA under the specified culture conditions. *n* = 3. **f** Flow cytometry analysis of UCB-CD34^+^-derived erythroid cells treated with DMSO or 2 μM GDC-0879 for 5 days and then subjected to cytokine deprivation (EPO, SCF and IL3) for 48 h, showing Annexin V-FITC (top) and PI (bottom) staining. **g** Whole-plate view of CFA with 200 UCB-CD34^+^ cells seeded in EPO-only Methocult H4430 and incubated under BRAFi (Encorafenib, GDC, or SB)-treated or control conditions for 14 days. Scale bar = 10 mm. **h** Statistical analysis of the area of 75 individual erythroid colonies in panel (**g**) on Day 14. Unless otherwise noted, all experiments used control (DMSO), SB-590885 at 1 μM, GDC-0879 at 2 μM, and Encorafenib at 0.5 μM. Error bars represent the mean ± SD. A two-tailed unpaired Student’s *t*-test was performed for the statistical comparison between two groups (****P < 0.0001)

Both SCF and EPO are essential for erythropoiesis through their role in MAPK activation, deficiency in either can result in ineffective erythropoiesis.^13–15^ Consequently, we investigated whether BRAFi could mitigate the attenuated MAPK pathway activation and the resulting ineffective erythropoiesis caused by SCF or EPO restriction. We found BRAFi impressively alleviated ineffective erythropoiesis by reducing the proportion of differentiation-arrested erythroid progenitors, thereby profoundly promoting erythroid differentiation (Fig. 2a–c, Supplementary Fig. 2a–d), enhancing hemoglobin synthesis, and decreasing the diameter of benzidine-positive cells (Fig. 2d, Supplementary Fig. 2e, f), indicative of erythroid maturation. Indeed, it is surprising to find that BRAFi treatment nearly rescued the inefficient differentiation process under SCF deprivation conditions (Fig. 2d, Supplementary Fig. 2a–d). Deprivation of SCF or reduction of EPO in erythroid cultures resulted in a marked decrease in total cell counts, with declines of 24.7-fold and 5.6-fold, respectively, compared to normal medium over a period of 14 days. However, the administration of GDC under SCF-deprived or EPO-reduced conditions substantially increased the cell counts—by 12.2-fold with 5% EPO and by 7.4-fold in SCF-free medium, compared to the respective untreated controls (Fig. 2e). Additionally, we found that BRAF inhibitor treatment reduced cytokine deprivation-induced apoptosis (Fig. 2f, Supplementary Fig. 2g). Moreover, the mean size of erythroid colonies increased by 3.4-fold with GDC and by 5.1-fold with SB, compared to the untreated control in methylcellulose medium containing only EPO (Fig. 2g, h). The above results suggest that BRAFi can effectively counteract ineffective erythropoiesis.

### BRAF inhibitors paradoxically activate ERK/MAPK during erythropoiesis

**Fig. 3:**
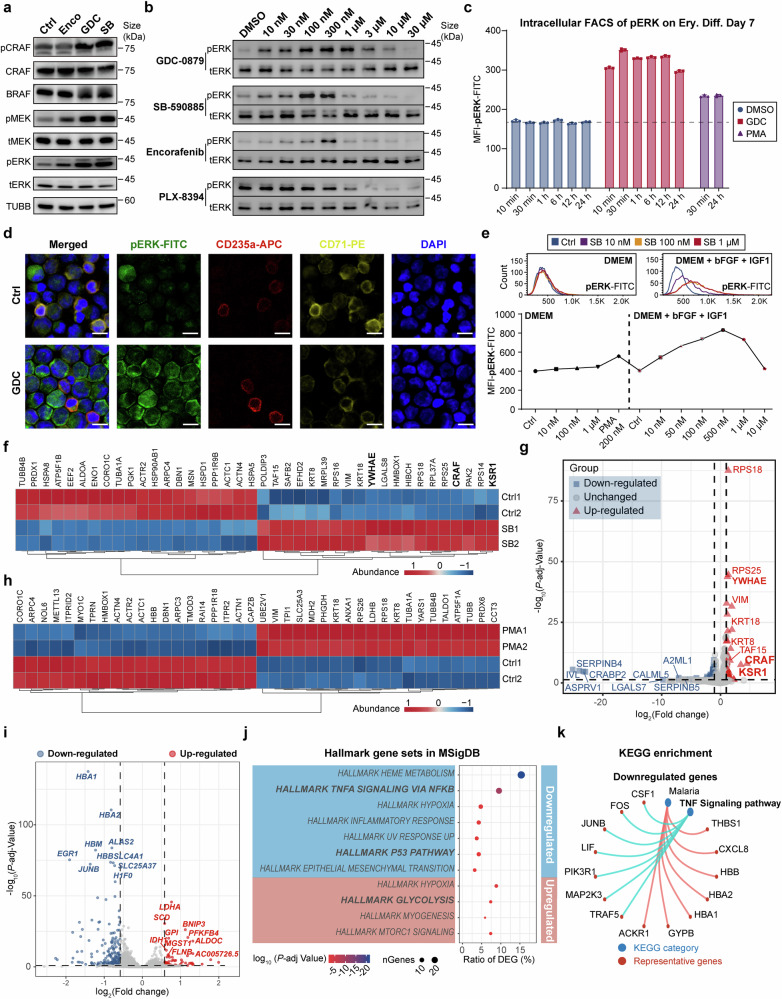
BRAF inhibitor-induced amplification of the ERK/MAPK cascade in erythroid progenitor cells was dependent on CRAF and the active extracellular signaling**. a** Immunoblotting of MAPK signaling cascade proteins in UCB-CD34^+^-derived erythroblasts cultured under normal conditions and treated on Day 9 with Encorafenib (0.5 μM), GDC-0879 (2 μM), or SB-590885 (0.5 μM) for 30 min. **b** Levels of phosphorylated and total ERK proteins in UCB-CD34^+^-derived erythroblasts on Day 9, cultured under normal conditions and treated with different BRAF inhibitors for 30 min. **c** Median fluorescence intensity (MFI) statistics of intracellular flow cytometry of phosphorylated-ERK-FITC of 7-day differentiated erythroid cells from UCB-CD34^+^ treated with 2 μM GDC or 200 nM PMA for different time period. The dashed line indicates the fluorescence intensity of the negative control. *n* = 3. **d** Immunofluorescence staining of phosphorylated-ERK-FITC, erythroid markers CD235a-APC and CD71-PE, and DAPI of 7-day differentiated erythroid cells from UCB-CD34^+^ treated for 30 min. Scale bar = 10 μm. **e** Representative intracellular flow cytometry histograms and MFI statistics of phosphorylated-ERK-FITC in 293 T cells under different culture system treated in different chemicals and concentration for 30 min. DMEM: DMEM with 10% FBS; bFGF (basic fibroblast growth factor), 50 ng/mL; IGF1 (insulin-like growth factor 1), 50 ng/mL. *n* = 3. **f** Heatmap of the top 20 proteins that were most significantly upregulated and downregulated respectively by 3 × Flag-BRAF interaction proteins in the control group (DMSO) and the 30-minute 1 μM SB-treated group, identified through flag-affinity immunoprecipitation-mass spectrometry (IP-MS) of 3 × Flag-BRAF in BRAF-overexpressing K562 cells. The components of the RAF protein dimer complex are bolded. cutoff, *p* < 0.05. **g** Volcano plot of 3 × Flag-BRAF interacting proteins in IP-MS of SB-treated and control groups in K562 cells. cutoff: *p-adj* < 0.05, foldchange > 2. The components of the RAF protein dimer complex are bolded. **h** Heatmap of the top 20 proteins that were most significantly upregulated and downregulated respectively by 3 × Flag-BRAF interaction proteins in the control group (DMSO) and the 30-minute 200 nM PMA-treated group, identified through Flag-affinity IP-MS of 3 × Flag-BRAF in BRAF-overexpressing K562 cells. cutoff, *p* < 0.01. **i** Volcano plot of the differentially expressed genes (DEGs) between CD71^+^ erythroid progenitor cells treated with 2 μM GDC for 72 h and control (DMSO) groups. DEGs were defined with a cutoff fold change > 1.5, FDR (false discovery rate) < 0.1. **j** The Molecular Signatures Database (MSigDB) hallmark gene sets enrichment analysis for DEGs between CD71^+^ erythroid progenitor cells treated with 2 μM GDC for 72 h and the control group. **k** Representative KEGG enrichment analysis of downregulated DEGs in the GDC-treated group compared to the control group. Error bars represent the mean ± SD

We next probed the mechanistic function of BRAFi in erythropoiesis. As previously reported, BRAFi are recognized to induce cell proliferation by activating the ERK/MAPK pathway in wild-type BRAF cells rather than blocking the MAPK cascade in BRAF^V600E^ mutant cells, a phenomenon known as “paradoxical activation”.^26–28^ All three BRAFi we tested paradoxically triggered MAPK signaling, leading to heightened phosphorylation of CRAF, MEK and ERK in erythroid progenitor cells in 30 min (Fig. 3a). In line with their capacity to enhance proliferation, GDC and SB triggered the most pronounced levels of ERK phosphorylation. Notably, GDC presented a wider effective concentration range (10–10,000 nM) for ERK activation compared to SB (10–300 nM), which aligns with its more extensive proliferation-promoting concentration spectrum. In contrast, PLX-8394, a “paradox breaker” and an analogue of Vemurafenib,^29,30^ neither increased cell numbers nor induced phosphorylation of ERK or MEK, as it is reported to circumvent the paradoxical MAPK pathway activation (Fig. 3b).

To investigate the time course of BRAFi activation in the MAPK pathway, we conducted intracellular flow cytometry staining for phosphorylated ERK in CD34^+^ cells that had undergone erythroid differentiation for 7 days. At all 6 treatment intervals, ranging from 10 min to 24 h, BRAFi treatment consistently and significantly elevated ERK phosphorylation levels across every erythroid cell subpopulation, including hematopoietic progenitors (CD71^−^CD235a^−^), erythroid progenitors (CD71^+^CD235a^−^) and precursors (CD71^+^CD235a^+^) (Fig. 3c, Supplementary Fig. 3a, b), and we also validated the upregulated ERK phosphorylation in erythroid cells through immunofluorescence staining (Fig. 3d). Notably, intracellular flow cytometry analysis revealed that the increase in pERK levels following GDC treatment was more pronounced in the erythroid progenitor stage (CD235a^-^) and less so in erythroid precursors (CD235a^+^), compared to DMSO-treated controls (Supplementary Fig. 3a, b). Generally, the extent of pERK elevation correlates with the level of RAS signaling activity during erythropoiesis, showing a decreasing trend from CD71^−^CD235a^−^ to CD71^+^CD235a^+^ cells. Interestingly, while the phorbol 12-myristate 13-acetate (PMA) is well-known for its strong ability to activate ERK/MAPK and induce megakaryocytic differentiation in K562 cells,^31^ its capacity to induce ERK phosphorylation in primary erythroid progenitor cells under these conditions was weaker than that of BRAFi (Fig. 3c, Supplementary Fig. 3b). Moreover, blocking MEK downstream or removing upstream cytokines to reduce RAS activation markedly impaired the paradoxical activation of ERK/MAPK (Supplementary Fig. 3c, d).

Given that the paradoxical activation of MAPK by BRAF inhibitors depends on cytokine-driven activation of upstream RAS signaling, we further investigated the necessity of active RAS signaling on BRAFi-induced upregulation of ERK phosphorylation. In regular 293 T cell cultures, BRAFi did not induce noticeable increase in pERK at any concentration, whereas PMA successfully induced pERK elevation. However, in the presence of additional insulin-like growth factor 1 (IGF1) and basic fibroblast growth factor (bFGF), while the control group showed no noticeable increase in pERK, BRAFi treatment for just 30 min resulted in a significant elevation in pERK levels, as revealed by intracellular flow cytometry (Fig. 3e, Supplementary Fig. 3e). Similarly, in the presence of EPO and IL3, BRAFi significantly induced pERK in K562 cells (Supplementary Fig. 3e, f). These results suggest that BRAF inhibitors do not directly activate ERK but rather amplify signaling from already activated RAS/MAPK pathways, leading to significantly increased ERK phosphorylation. During erythroid differentiation under BRAFi treatment, each cytokine alone could trigger paradoxical activation, although the extent of ERK phosphorylation varied depending on the specific cytokine (Supplementary Fig. 3g).

Furthermore, knocking down different RAF proteins in human CD34^+^ erythroid cultures revealed that targeting CRAF led to a significant reduction in both cell proliferation and MAPK pathway activation (Supplementary Fig. 3h–j). These findings underscore the direct impact of BRAFi on ERK/MAPK signaling in erythroid cells.

Previous studies have shown that the paradoxical activation of MAPK by BRAF inhibitors is primarily dependent on the dimerization of RAF proteins, particularly with CRAF.^29,32–34^ To further explore the differences in BRAF-interacting proteins induced by BRAF inhibitor treatment, we overexpressed 3 × Flag-BRAF in K562 cells and conducted co-immunoprecipitation followed by mass spectrometry (IP-MS). Overall, the BRAFi-treated group exhibited a downregulation of most BRAF-interacting proteins, whereas the PMA-treated group showed an upregulation (Supplementary Fig. 4a). Notably, BRAF inhibitor treatment led to a substantial increase in BRAF’s interaction with CRAF (6.04-fold), KSR1 (7.38-fold), and YWHAE, all of which are key components involved in RAF dimerization and scaffolding (Fig. 3f, g). Conversely, PMA treatment enhanced the interaction between BRAF and microtubules (e.g., TUBB, TUBB4B and TUBA1A), whereas this interaction was noticeably diminished in the BRAFi treatment group (Fig. 3f, h). Immunoblotting analysis confirmed that BRAF’s interaction with CRAF significantly increased under BRAFi treatment. In contrast, PMA treatment enhanced BRAF’s interaction with ARAF, illustrating the distinct interaction profiles induced by different treatments, despite both leading to ERK phosphorylation (Supplementary Fig. 4b).

Furthermore, RNA-seq analysis of sorted CD71^+^ erythroid progenitor cells treated with GDC for 72 h showed a relatively modest impact on the transcriptome; only 189 downregulated and 68 upregulated genes were found by a cutoff of a fold change > 1.5 (Fig. 3i). The treated cells exhibited an enrichment of biological processes including glycolysis and carbon metabolism while down-regulating P53 and TNF activation (Fig. 3j, k, Supplementary Fig. 4c). GDC treatment downregulated genes involved in erythropoiesis and hemoglobin biosynthesis, while upregulating genes associated with the cell cycle, such as *MYC* and *CDK6*, thus supporting cell proliferation and delaying the maturation process (Supplementary Fig. 4d).

In summary, these findings indicate that BRAFi stimulate erythroid proliferation through paradoxically activating the ERK/MAPK pathway, a process that depends on cytokines and CRAF.

### Constitutive activation by BRAF^V600E^, unlike BRAF inhibitors, disrupted hematopoietic stem cell maintenance and abolished erythropoiesis

While the BRAF^V600E^ mutation is predominantly associated with a high incidence of mutation in solid tumors, such as melanoma, it has also been identified in various hematological malignancies.^35–37^ For instance, LCH, which is a rare disorder characterized by the aggressive proliferation of CD1a^+^ Langerhans cells originating from myeloid progenitors, often exhibits the BRAF^V600E^ mutation.^38–40^ Prior studies have shown that HSPCs harboring the BRAF^V600E^ mutation undergo abnormal MAPK activation, leading to disrupted erythropoiesis,^35,38,39^ a process that differs markedly from the paradoxical activation triggered by BRAFi in erythropoiesis. Therefore, we are keen to elucidate the mechanistic distinctions underlying the contrasting erythroid phenotypes in HSPCs caused by paradoxical activation through BRAFi versus the constitutive activation triggered by BRAF^V600E^ both activating ERK, but leading to contradictory phenotypes.

**Fig. 4:**
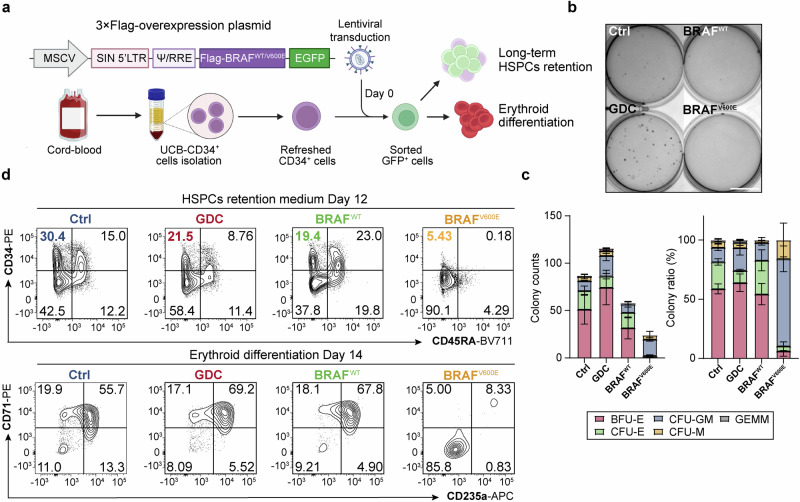
BRAF^V600E^, rather than BRAF inhibitors, disrupted hematopoiesis and erythropoiesis**. a** Schematic diagram of lentiviral transduction and culture strategy of primary human UCB-CD34^+^ HSPCs. **b** Representative images of 300 HSPCs pre-cultured for 2 days in erythroid differentiation medium before seeded into Methocult H4435. **c** Corresponding colony counts and lineage ratios calculated on Day 14 of panel (**b**). Scale bar = 10 mm. **d** Flow cytometry analysis of HSPC surface markers on Day 12 in HSPC retention medium (top), and erythroid cell surface markers on Day 14 in erythroid differentiation medium (bottom), following lentiviral transduction or drug treatment. The control (Ctrl) and GDC groups were transduced with MSCV-GFP empty vector and treated with DMSO or 2 μM GDC-0879, respectively

We first overexpressed the BRAF^V600E^ mutant and wild-type BRAF in human UCB-CD34^+^ HSPCs to examine their impact on stem cell maintenance and erythroid differentiation (Fig. 4a). Colony-forming assays showed that BRAFi modestly promoted erythroid colony formation, while BRAF^V600E^ overexpression nearly completely inhibited erythroid colony formation, without impacting the number of granulocytes and monocytes colonies (Fig. 4b, c), suggesting BRAF^V600E^ did not result in a HSPC lineage skewing towards other myeloid lineages. Then we found cells expressing BRAF^V600E^ exhibited a rapid decline in the HSPC population in HSPCs retention medium, marked by elevated levels of dendritic cell (CD11c) and monocyte/macrophage (CD11b) markers (Fig. 4d, Supplementary Fg. 5a–d), which was not observed in the other groups, particularly the GDC-treated group.

In UCB-CD34^+^ cell erythroid differentiation culture, BRAF^V600E^ overexpression drastically impeded erythropoiesis (Fig. 4d), while wild-type BRAF overexpression appeared to delay erythroid differentiation. Similarly, GDC treatment decelerated the differentiation process while promoting proliferation, consistent with the results above (Fig. 4d, Supplementary Fig. 5e, f). Interestingly, BRAF^WT^ overexpression did not affect cell proliferation or interrupt the pro-proliferative effect of BRAFi (Supplementary Fig. 5e, f).

These findings suggest that overexpression of BRAF^V600E^ nearly eradicated erythropoiesis in HSPCs and blocked the proliferation and differentiation of the erythroid lineage, a scenario not observed with BRAF inhibitors.

### BRAF^V600E^ disrupted hematopoiesis and erythropoiesis by hyperactivating AP-1 family transcription factors

**Fig. 5:**
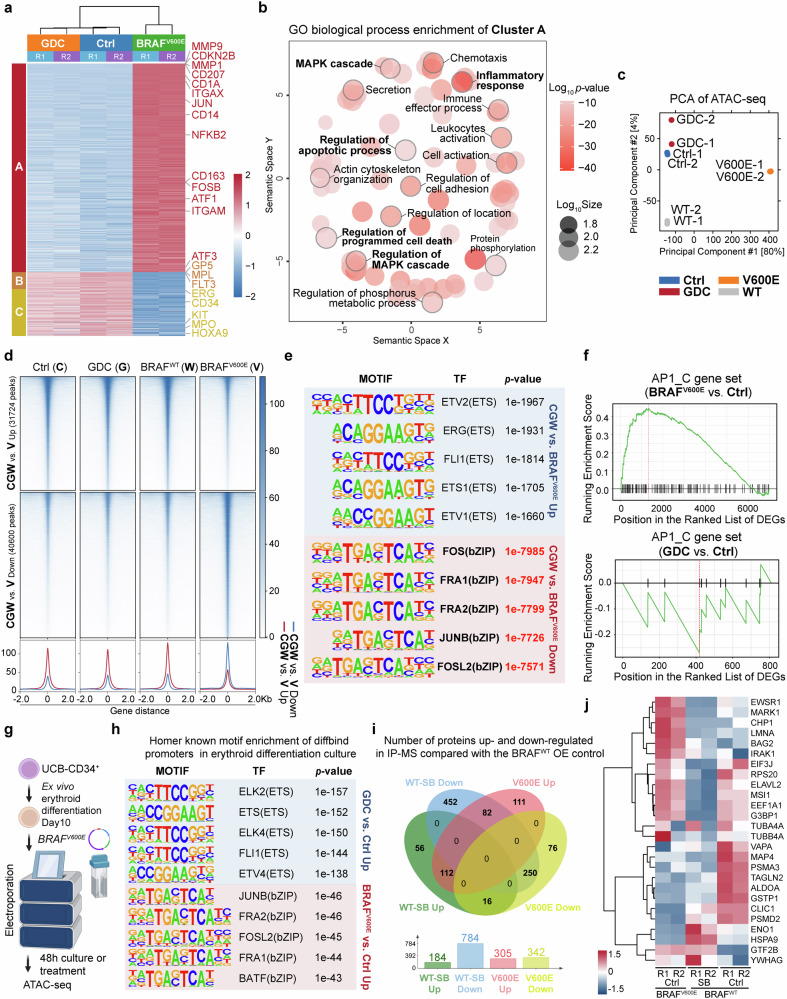
BRAF^V600E^, rather than BRAF inhibitors, disrupted hematopoiesis and erythropoiesis via hyperactivating AP-1 family transcription factors**. a** K-means clustering heatmap of the top 2000 most variable genes in UCB-CD34^+^ cells across 3 groups on Day 6 of treatment/transduction in HSPCs retention medium. **b** Semantic plot of the GO terms enriched in gene cluster A from panel (**a**), generated using online tool GENE ONTOLOGY (GO) TOOLS and REVIGO (http://revigo.irb.hr/) to illustrate the similarity among non-redundant GO terms. Bubble color represents the log10(*p*-value), and bubble size reflects the geneset size of the GO term in the Gene Ontology Biological Process (GOBP) database. Representative GOBP terms are in bold font, with the bubbles of the corresponding processes outlined in light grey. **c** Principal component analysis (PCA) of ATAC-seq data on UCB-CD34^+^ cells on Day 6 of treatment or lentiviral transduction in HSPCs retention medium. WT, BRAF^WT^; V600E, BRAF^V600E^. **d** Differential chromatin accessibility peaks in the UCB-CD34^+^ ATAC-seq analysis, comparing the Ctrl (DMSO), GDC and BRAF^WT^ (CGW) group’s common region to the BRAF^V600E^ (V) group. The value 0 represents the peak center in ATAC-seq. **e** Known motif enrichment analysis of differential ATAC-seq peaks in genome from the CGW group and the BRAF^V600E^ group using HOMER software. The table displays top transcription-factor-binding motifs enriched in the ATAC-seq data of the CGW group (Top, blue shade) and the BRAF^V600E^ group (Bottom, red shade). **f** Gene Set Enrichment Analysis (GSEA) analysis of the AP-1 family gene set (AP1_C) in DEGs comparing BRAF^V600E^ vs. control groups and GDC vs. control groups. **g** Schematic diagram of the experimental workflow for electroporation BRAF^V600E^ and ATAC-seq of 10-Day erythroid differentiated UCB-CD34^+^ cells. **h** Known motif enrichment analysis of differentially binding (diffbind) ATAC-seq peaks of promoter region between the GDC group and the BRAF^V600E^ group cells compared with control group in panel (**g**) using HOMER software. The table displays top enriched transcription-factor-binding motifs in the ATAC-seq data of the GDC group (Top, blue shade) and the BRAF^V600E^ group compared with control group (Bottom, red shade). **i** Number of proteins up- and down-regulated in Flag-affinity IP-MS in BRAF^WT^ overexpressing cells treated with 1 μM SB for 30 min, or in BRAF^V600E^ overexpressing 293 T, compared to the BRAF^WT^ overexpressing group. Protein cutoff foldchange > 1.5. **j** Heatmap showing the abundance of AP-1-associated proteins (GSEA gene set AP1_C) interacting with Flag-BRAF^WT^ (with or without BRAFi) or Flag-BRAF^V600E^ in IP-MS. For ATAC-seq and RNA-seq, both the control and GDC groups were transfected with MSCV-GFP empty vector and treated with DMSO or 2 μM GDC-0879, respectively

To understand the divergent phenotypes resulting from BRAF^V600E^ overexpression compared to BRAFi treatment, we conducted RNA-seq analysis on HSPCs overexpressing BRAF^V600E^, and control, with or without GDC treatment. K-means clustering of the most variable 2000 genes yielded three clusters. Cluster A, which included 1532 genes, such as *MMP9*, *MMP1*, *CD207*, *CD1A*, *CD11C* (*ITGAX*), and *JUN*, characterized a CD1a^+^ Langerhans cells phenotype with pathway enrichment in the MAPK cascade and inflammatory response, indicating a dendritic cell-like expression profile consistent with the reported BRAF^V600E^ phenotype (Fig. 5a, b, Supplementary Fig. 6a).^35,38^ Clusters B and C, containing genes such as *MPL*, *FLT3*, *ERG*, *CD34*, *MPO*, and *KIT* associated with HSPC maintenance, were enriched in the control and GDC groups, suggesting that BRAF^V600E^ disrupts genes crucial for sustaining HSPCs (Fig. 5a, Supplementary Fig. 6b, c). Unlike BRAF^V600E^, BRAFi treatment only slightly affected the transcriptome, it nevertheless promoted cell cycle progression and various RNA-related processes (Supplementary Fig. 6d, e).

ATAC-seq analysis revealed distinct chromatin accessibility patterns in the BRAF^V600E^ group, differing from the control, GDC-treated, and BRAF^WT^ groups in HSPC retention culture system (Fig. 5c, d). In comparing BRAF^V600E^ to the common peaks found in the control, GDC, and BRAF^WT^ (CGW) group, motif analysis from whole genome and promoters both underscored a significant enrichment (genome, *p*-value < 1e^−7000^) of AP-1 family transcription factors in BRAF^V600E^-specific peaks, while the peaks for the control, GDC, and BRAF^WT^ groups were associated with ETS family motifs, known for their roles in hematopoietic maintenance and differentiation (Fig. 5d, e, Supplementary Fig. 6f).

Integrating transcriptome profiling, it was observed that BRAFi modestly activated genes related to proliferation, including *MYB*, *CDK2, CDK6*. Conversely, overexpression of BRAF^V600E^ markedly increased the overall expression of AP-1 family transcription factors and related genes such as *JUN*, *FOS*, and *JUNB*, which did not show significant upregulation with BRAFi treatment (Supplementary Fig. 7a, b). Furthermore, Gene Set Enrichment Analysis (GSEA) confirmed that BRAF^V600E^ induced the activation of pathways associated with AP-1, MAPK and a BRAF mutant signature like hairy cell leukemia (Fig. 5f, Supplementary Fig. 7c, d). Increased chromatin accessibility and higher expression levels of *JUN* and *FOS* were also evident in the BRAF^V600E^ group (Supplementary Fig. 7e). Interestingly, RNA-seq analysis revealed that BRAF inhibitors even had the opposite effect, downregulating *JUNB* and *FOS* expression in the UCB-CD34^+^ erythroid differentiation system (Fig. 3i, k).

To further validate whether AP-1 activation is also induced by BRAF^V600E^ in erythroid progenitor cells, we overexpressed BRAF^V600E^ or administered BRAFi, followed by ATAC-Seq analysis on CD34^+^ cells on Day 10 of erythroid differentiation. Consistently, our results showed that the BRAF^V600E^ group exhibited enrichment of AP-1-related motifs in the genomic promoter regions compared to the control group, while the BRAF inhibitor-treated group exhibited enrichment of ETS family motifs (Fig. 5g, h). This evidence further supports the distinct effects of these two BRAF activation scenarios on transcription factor activation and genomic targeting.

Finally, to investigate potential differences in interacting proteomics between BRAF^WT^ in the presence of a BRAF inhibitor and BRAF^V600E^, we conducted IP-MS using 3 × Flag-BRAF^WT^ and 3 × Flag-BRAF^V600E^ overexpressing 293 T cells. Consistent with the findings in K562 cells, the abundance of BRAF^WT^-interacting proteins decreased following BRAF inhibitor treatment (Fig. 5i, Supplementary Fig. 7f). The SB-treated group exhibited stronger interactions with ribosomal proteins (RPL27A, RPS18, and RPS11) and BRAF compared to the BRAF^V600E^ group. Interestingly, the BRAF^V600E^ group demonstrated more interactions with eukaryotic translation initiation factors (EIF3A, EIF3F, and EIF3E) (Supplementary Fig. 7g). Additionally, the BRAF inhibitor-treated group showed reduced interactions with AP-1 related proteins compared to the BRAF^V600E^ and BRAF^WT^ control groups (Fig. 5j).

Collectively, our multi-omics analyses reveal that the preferential interaction of AP-1 with BRAF^V600E^ provides insights into the distinct erythroid and HSPC phenotypes induced by paradoxical BRAFi activation versus BRAF^V600E^’s constitutive activation, despite both leading to ERK/MAPK activation.

### In vivo effects of BRAFi on human hematopoiesis, erythroid differentiation, and protection against cisplatin-induced myelosuppression in mouse models

**Fig. 6:**
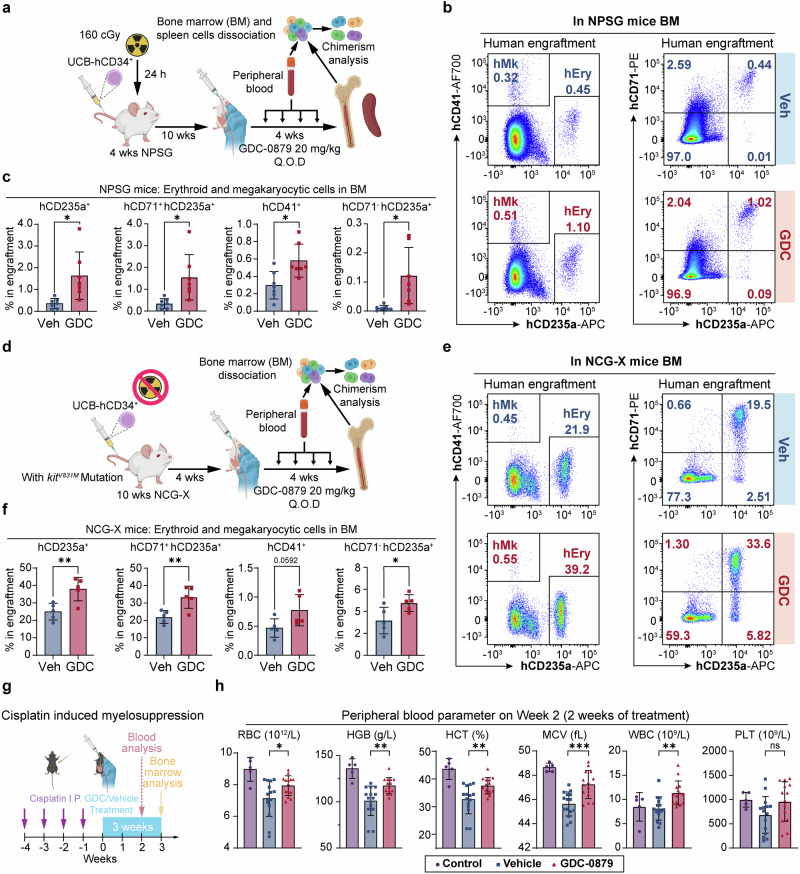
BRAF inhibitors promoted erythroid and megakaryocytic engraftment in human hematopoietic reconstitution, and alleviated anemia in vivo*.* **a** Schematic diagram of the human hematopoietic reconstitution model in irradiated NPSG mice. Q.O.D., every other day. **b** Representative flow cytometry analysis of the proportion of erythroid cells and megakaryocytes in human engraftment and erythroid differentiation in the bone marrow of NPSG mice 14 weeks after transplantation. **c** Statistics on the proportion of erythroid cells and megakaryocytes in human engraftment in NPSG mice bone marrow 14 weeks after transplantation. *n* = 7 in each group in NPSG model. Each dot represents one mouse. **d** Schematic diagram of the human hematopoietic reconstitution model in irradiated-free NCG-X mice (**e**). Representative flow cytometry analysis of the proportion of erythroid cells and megakaryocytes in human engraftment and erythroid differentiation in the bone marrow of NCG-X mice 8 weeks after transplantation. **f** Statistics on the proportion of erythroid cells and megakaryocytes in human engraftment in the bone marrow of NCG-X mice 8 weeks after transplantation. *n* = 5 in each group in NCG-X mice model. Each dot represents one mouse. **g** Schematic diagram of GDC administration in the cisplatin-induced myelosuppression model. Purple arrows indicate cisplatin induction; blue shaded box indicates duration of treatments (vehicle or GDC). Blood cell parameters were measured in Week 2 and bone marrow cell composition was analyzed in Week 3 through flow cytometry. Each dot represents one mouse. **h** Red blood cell parameters were measured from mouse peripheral blood after 2 weeks of treatment. For the control group, *n* = 5; for the vehicle group, *n* = 15; for the GDC group, *n* = 14. Each dot represents one mouse. Error bars represent the mean ± SD. A two-tailed unpaired Student’s *t*-test was performed for the statistical comparison between two groups (ns, *P* > 0.05; *P < 0.05; **P < 0.01; ***P < 0.001)

Given the observed enhancement of human erythropoiesis by BRAFi in vitro, we next examined their effects on human hematopoiesis and erythroid differentiation in vivo. Human UCB-CD34^+^ HSPCs were transplanted into NPSG mice for 10 weeks, then followed by a 4-week administration of GDC (Fig. 6a). We observed a significant increase in the percentage of human hematopoietic cells (hCD45^+^) in the peripheral blood during GDC treatment (Supplementary Fig. 8a, b). At 14 weeks post-transplantation, while most lineage proportions and overall human engraftment in the bone marrow were similar (Supplementary Fig. 8c, d), there was a marked increase in the ratios of human erythroid cells (from 0.36% to 1.63%) and megakaryocytes (from 0.3% to 0.58%) (Fig. 6b, c). Additionally, there was a more advanced erythroid differentiation pattern, as indicated by an increase in terminally differentiated erythroblasts (hCD71^−^hCD235a^+^) (Fig. 6c). The HSPC (hCD45RA^−^ hCD34^+^) ratio remained similar, but there was an uptick in the progenitor cell (hCD34^+^) population in the GDC-treated group (Supplementary Fig. 8e). Additionally, human engraftment in the spleen increased after GDC treatment, yet no human erythroid reconstitution was detected in the spleen (Supplementary Fig. 8f).

Since NPSG mice typically exhibit low erythroid engraftment efficiency, making them less than ideal for our model, we repeated HSPC transplantations in the NCG-X model. The NCG-X model harbors an additional *Kit* mutation compared to NPSG mice, enabling higher and faster human erythroid reconstitution efficiency without the need for irradiation (Fig. 6d).^21^ Consistent with previous experiments, there was a higher ratio of human CD45^+^ cells in the peripheral blood of NCG-X mice during the 4 weeks of GDC treatment (Supplementary Fig. 9a). Besides, there was a significant elevation in the percentage of erythroid cells and a moderate increase in the percentage of megakaryocytes within the human graft in the bone marrow (Fig. 6e, f). This occurred alongside comparable levels of human engraftment and lineage distribution in the bone marrow (Supplementary Fig. 9b, c). The proportions of human HSPCs in the bone marrow were similar in the GDC group (Supplementary Fig. 9d).

Furthermore, we evaluated the effects of GDC on a mouse model of cisplatin-induced myelosuppression (Fig. 6g), which commonly results in anemia.^41^ Treatment with GDC mitigated this condition, as evidenced by improved red blood cell parameters in the peripheral blood (Fig. 6h) and increased bone marrow cellularity comparable to the vehicle group. Interestingly, GDC treatment not only ameliorated the anemic phenotype in peripheral blood but also reversed the anemia-induced erythropoiesis shift in the bone marrow, restoring it to normal levels comparable to the control group without modeling, with an enhanced population of CD71^+^ Ter119^+^ erythroblasts (Supplementary Fig. 9e, f). An additional in vivo experiment demonstrated the protective effects against cisplatin-induced myelosuppression, showing improved blood cell parameters (Supplementary Fig. 9g). Together, these results provide evidence that BRAF inhibitors promote human hematopoietic and erythroid development in mouse models, while also highlighting the potential of BRAFi to alleviate anemia in vivo.

### BRAF inhibitors improved erythroid differentiation from Diamond-Blackfan anemia patient-derived cells in vitro and alleviated anemia in the *Rpl11* haploinsufficiency mouse model in vivo

**Fig. 7:**
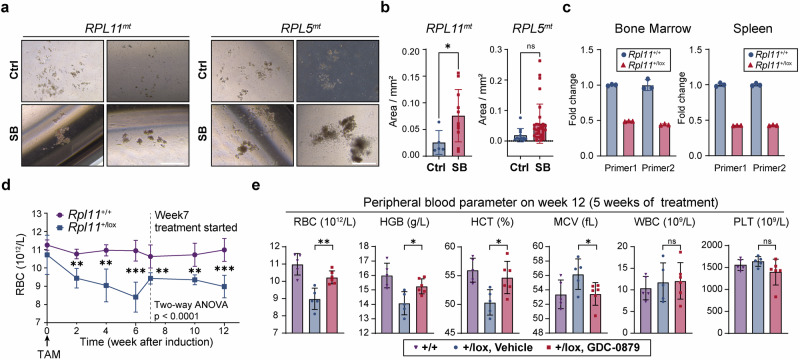
BRAF inhibitors improved erythroid proliferation in cells from DBA patients in vitro and alleviated anemia in *Rpl11* haploinsufficiency mice in vivo. **a** Representative images of erythroid colonies derived from 10^5^ PBMCs of DBA patients with *RPL5* or *RPL11* mutations, seeded in Methocult H4435 for 14 days. **b** Quantification of erythroid colony area from DBA patients’ PBMCs, as shown in panel (**a**) on Day 14. **c** *Rpl11* mRNA levels measured by qRT-PCR in bone marrow and spleen using 2 individual primer pairs in *Rpl11*^+/lox^ (21 weeks after TAM induction) and *Rpl11*^+/+^ mice. *Gapdh* mRNA levels were used as internal control. Each dot represents a technical replicate, *n* = 3. **d** Red blood cell (RBC) count for *Rpl11*^+/+^ and *Rpl11*^+/lox^ (the +/lox vehicle group in panel (**e**)) mice after the initiation of TAM induction at week 0. The dashed line indicates that the *Rpl11*^+/lox^ group was administered either vehicle or GDC started from week 7. The asterisks represent the results of the *t*-test between the two groups at each time point. The *p*-value from the two-way ANOVA for the comparison between the two groups is also shown in panel (**d**). **e** RBC count, hemoglobin (HGB), hematocrit (HCT), mean corpuscular volume (MCV), white blood cell (WBC) and platelet (PLT) count were measured after 5 weeks of GDC administration in the *Rpl11* haploinsufficiency DBA mouse model (*n* = 5 or 6). Each dot represents one mouse. Error bars represent the mean ± SD. A two-tailed unpaired Student’s *t*-test was performed for the statistical comparison between two groups (ns, *P* > 0.05; *P < 0.05; **P < 0.01; ***P < 0.001; ****P < 0.0001)

Human disorders that affect erythroid development can stem from either the disrupted proliferation of erythroid progenitors or impaired erythroid differentiation and maturation. Diamond-Blackfan anemia (DBA) is a genetic bone marrow failure, typically associated with mutations in genes responsible for ribosome biogenesis.^7,8^ Less than half of the patients respond to steroids, the primary treatment option, and the only definitive cure is hematopoietic stem cell transplantation.^7,8,42^ In our study, we observed that treatment with BRAFi led to the formation of larger erythroid colonies in PBMCs from DBA patients with *RPL5* or *RPL11* mutations (Fig. 7a, b).

The *Rpl11* haploinsufficiency DBA mouse model, reported to recapitulate macrocytic anemia and erythroid marrow failure characteristic of the disorder,^43,44^ was validated in our studies (Fig. 7c, d). After 5 weeks of GDC treatment, pronounced improvements in erythroid parameters (RBC and HGB) were observed, effectively reversing the macrocytic anemia phenotype without impacting WBC and PLT counts (Fig. 7e).

**Fig. 8:**
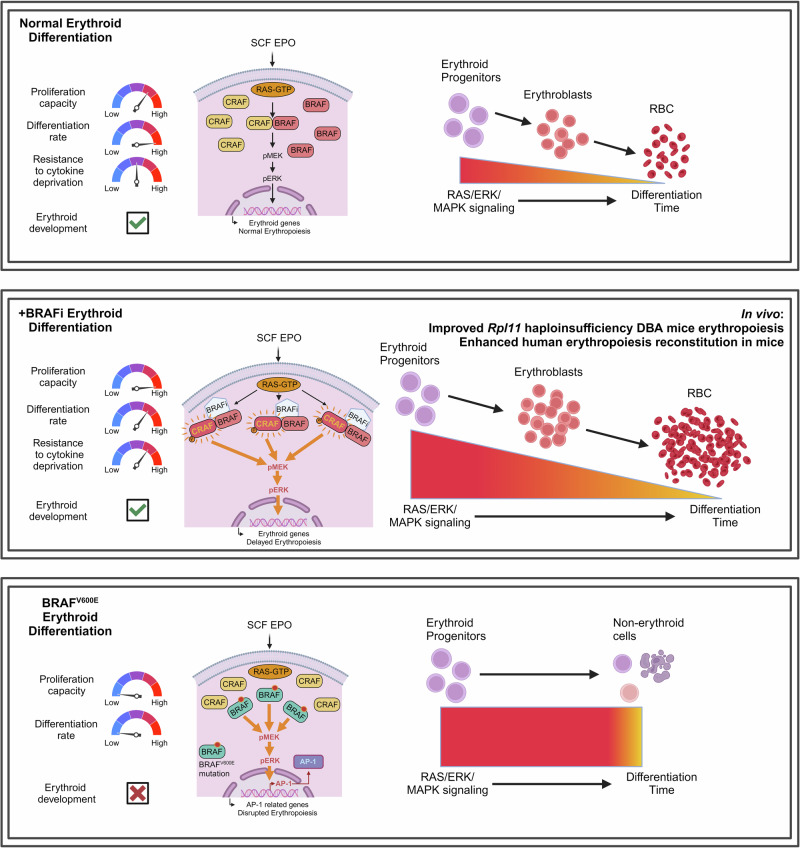
Graphic summary. (Top Panel: Normal Erythroid Differentiation) SCF and EPO activate the RAS/ERK/MAPK pathway, supporting and balanced erythroid progenitor proliferation, differentiation, and normal RBC production. (Middle Panel: BRAFi-Enhanced Erythroid Differentiation) BRAF inhibitors (BRAFi) paradoxically amplify RAS/ERK/MAPK signaling, promoting erythroid progenitor proliferation and temporarily delaying differentiation, leading to increased RBC production. (Bottom Panel: BRAF^V600E^-Mediated Erythroid Disruption) The constitutive activation of BRAF (BRAF^V600E^ mutation) disrupts erythroid differentiation, leading to abnormal cell fate decisions and impaired erythropoiesis due to excessive MAPK signaling and hyperactivation of AP-1. Graphic summary was generated with BioRender (https://app.biorender.com/)

In summary, our study highlights the potential value of BRAF inhibitors in treating anemia, from their therapeutic effects observed in vitro to their efficacy in DBA animal models, offering promise for improving outcomes in patients with refractory anemia and related disorders (Fig. 8).

## Discussion

While EPO has been clinically effective over the past two decades, certain anemias, including those caused by hemolysis and genetic bone marrow failure disorders like DBA, do not respond well to EPO treatment due to a scarcity and functional deficiency of erythroid progenitor cells.^4,8^ Newly approved drugs like HIF-PHD inhibitors and activin receptor ligand traps are dependent on the late erythroid progenitors for efficacy.^45,46^ Our understanding of the mechanisms driving BFU-E cells self-renewal and the development of drugs to enhance their proliferation remains incomplete. To address this, we devised an efficient primary human UCB-CD34^+^ HSPC-based screening system that identified erythropoiesis-promoting agents with a single chemical application and a 7-day culture period for robust erythroid differentiation, facilitating straightforward high-throughput proliferation assessment. We uncovered BRAF inhibitors as promising agents for boosting erythroid progenitor cell proliferation and differentiation, paving the way for novel treatments for a variety of anemia conditions, including DBA.

RAS signaling in erythroid progenitors is synergistically activated by SCF and EPO and is naturally down-regulated during differentiation, making erythropoiesis an ideal scenario to harness the pro-proliferative effects of BRAFi via amplifying RAS/MAPK signaling.^15,16^ These inhibitors work by enhancing the ERK/MAPK pathway in erythroid cells with wildtype RAS activation, especially initiated by essential external cytokines for cells, thereby extending the proliferation window with limited adverse effects on maturation or enucleation. Our intracellular flow cytometry experiments in primary erythroid progenitor cells, as well as in 293T and K562 cell lines, demonstrate that BRAF inhibitors can potently amplify MAPK signaling in a manner highly dependent on the presence of cytokines, whereas cytokine addition alone did not elicit such significant increase in MAPK activity. Moreover, we highlight that the role of BRAF inhibitors as signaling amplifiers is distinct from that of phorbol esters. The IP-MS results support the conclusion that BRAFi facilitates BRAF and CRAF dimerization while reducing the number and levels of overall BRAF protein interactions, presenting a proteomic landscape distinctly different from that observed with PMA treatment.

Contrarily, the BRAF^V600E^, a primary cause of hematological diseases like Langerhans cell histiocytosis and hairy cell leukemia and the target of BRAFi—leads to constitutive MAPK pathway activation, and further HSPC dysfunction, and erythropoiesis impairment, with a shift towards mononuclear/dendritic differentiation.^35,38,39^ While the exact mechanisms of BRAF^V600E^-induced LCH in HSPCs are still being decoded, it is known to trigger a senescence program in early multipotent HSPCs.^35,36,39^ Our study recapitulated the previously reported BRAF^V600E^ phenotype and further indicated AP-1 as a potential primary driver. Unlike the mutation, BRAFi promote hematopoiesis without biasing towards monocyte differentiation or significant stemness loss. This difference may arise from the persistent activation of oncogenic mutations, leading to continuous de novo signaling and variations in proteins interacting with BRAF^V600E^. In contrast, BRAFi pharmacologically amplifies and prolongs the typical activation signal. During erythroid development, RAS activation in the MAPK pathway is regulated by the reduced expression of upstream factors and receptors like KIT and EPOR. As a result, the amplified MAPK phosphorylation induced by BRAF inhibitors remains under the control of normal developmental processes, thereby minimizing side effects.

Since 2010, the paradoxical activation of BRAFi has been recognized and well-documented.^26–28^ Studies in structural biology evidence reveal that this unique effect stems from the chemical structure of RAF inhibitors, which stabilizes the αC-helix in either the IN or OUT conformations, facilitating the formation of naturally activated or suboptimal RAF dimers, particularly between BRAF and CRAF.^32,33,47,48^ The mass spectrometry results from our study not only confirmed the role of BRAF in the MAPK cascade and its activation through dimerization with CRAF but also identified other interacting proteins potentially impacted by BRAF inhibitor treatment, such as ribosomal proteins RPL27A, RPS18, RPS25, and RPL37A. It might be worthwhile for future studies to explore whether BRAF has additional yet undiscovered roles in ribosomopathies like Diamond-Blackfan anemia. Moreover, the enhanced interaction between BRAF^V600E^ and translation initiation factors hints at a possible link between BRAF and the regulation of translation.

RAF kinase inhibitors are classified based on the kinase conformation they induce, particularly the position of the αC-helix, a highly conserved structural element, into primarily “αC-IN” and “αC-OUT” types. “αC-IN” inhibitors, particularly “type-I” inhibitors which induce both α-helixes-IN and DFG motif-IN conformation of BRAF, such as GDC-0879 and SB-590885, enhance the interaction between RAF and RAS-GTP, promoting the formation of the RAF-MEK complex. On the other hand, “αC-OUT” inhibitors, such as vemurafenib and dabrafenib, have important clinical implications and have been observed to induce less paradoxical activation, likely due to decreased RAF/MEK interaction in this conformation.^29,30,33,48^ Although paradoxical activation limits the use of type I BRAFi in cancer therapy, their role is beneficial in scenarios where this activation is desired, such as wound healing,^49^ promoting podocytes survival,^50^ and here, erythropoiesis.

Taken together, our study demonstrates that BRAFi could effectively promote erythroid regeneration across various models of erythropoiesis disorders. Given their robust pro-proliferation effect, favorable safety profile, and the natural downregulation of RAS during erythropoiesis, BRAFi present as promising candidates for treating erythroid aplasia or related diseases with a low risk of adverse effects. Future exploration of BRAFi as an effective ERK amplifier or favorable activator under specific circumstances may further illuminate its potential as a regenerative agent in vitro or as a therapeutic for certain pathological conditions.

## Materials and methods

Reagents, antibodies, shRNAs and other details and information of experiments are provided in Supplementary Materials.

### Ethics statements

Peking University Ethics Committee, Ethics Committee of Capital Medical Affiliated Beijing Children’s Hospital approved the collection of samples from healthy donors, and the DBA patients. We received informed consent from all volunteers and patients, conforming to the Declaration of Helsinki. All animal experiments were performed under the Animal Protection Guidelines of Peking University, China, and all animal procedures were approved by the Ethical Committee of Peking University (LSC-LiXY-01/02) and the Ethical Committee of Westlake University (AP#22-009-GXF).

### Plasmids

Lentivirus overexpression vector was constructed based on pMSCV-3×Flag-T2A-GFP, including pMSCV-3×Flag-BRAF^WT^-T2A-GFP and pMSCV-3×Flag-BRAF^V600E^-T2A-GFP.

### Cell lines

Human embryonic kidney epithelial cell line HEK293T and chronic myelogenous leukemia cell line K562 were purchased from ATCC and authenticated by short tandem repeat identification. HEK293T was maintained in DMEM (Gibco, C1195500BT) supplemented with 10% fetal bovine serum (FBS) (ExCell, FSP500) and K562 was maintained in RPMI-1640 (Gibco, C11875500BT) with 10% FBS. In the intracellular flow cytometry of drug treatment, 293T cells were supplemented with 50 ng/mL IGF1 (PeproTech, 100-11) and 50 ng/mL bFGF (PeproTech, 100-18B). K562 were supplemented with 10 IU/mL EPO (Amgen, 55513-144-10) and 20 ng/mL IL3 (StemCell Technologies, 78042).

### UCB-CD34^+^ cells

Umbilical cord blood derived-human CD34^+^ cells (UCB-derived CD34^+^ cells): Human hematopoietic stem/progenitor cells (HSPCs, UCB-CD34^+^ cells) were purified from cord blood samples obtained from the Cord Blood Bank of Beijing. The erythroid culture medium is based on IMDM, and further supplemented with 10% FBS (Gibco, 10099141), 300 μg/mL human holo-transferrin (Sigma, T0665), 5% human AB serum (Wokavi Biotech, Beijing), 10 ng/mL heparin (Sigma, H3149), 10 μg/mL insulin (Sigma, I9278), 2 mM L-glutamine (Gibco, 25030081), 3 IU/mL erythropoietin (Amgen, 55513-144-10), 50 ng/mL SCF (StemCell Technologies, 78062), and 10 ng/mL IL3 (StemCell Technologies, 78042).

### Cell transfection

For BRAF overexpression in 293 T cells, plasmids were transfected using lipo8000 (Beyotime, C0533). For erythroid cells, BRAF^V600E^ overexpression was achieved by nucleofecting plasmids into CD34^+^ cells that had undergone 10 days of erythroid differentiation, following the manufacturer’s protocol. In brief, 10^6^ erythroid cells were spun down and resuspended in solution P3 for primary cells (LONZA, V4XP-3024). Plasmids of pMSCV-GFP (Empty vector) or pMSCV-3 × Flag-BRAF^V600E^-T2A-GFP were then added to the cell suspension, and nucleofection was carried out using a 4D-Nucleofector X Unit (Lonza Bioscience, TX, USA) with program EO-100. The pMSCV-GFP vehicle vector was used for both the Ctrl and the GDC-treated groups.

### Chemicals

GDC-0879 (MCE, HY-50864), SB-590885 (MCE, HY-10966) and Encorafenib (MCE, HY-15605) were dissolved in DMSO for in vitro culture. For in vivo experiments, GDC-0879 was dissolved in 50% PEG-300 and 50% PBS at concentrations of 2, 3, and 5 mg/mL.

### Cell proliferation assay

Cell proliferation was assessed by a Luna automated fluorescence cell counter L20001 (Logos BioSystems, South Korea) or by the CellTiter-Blue assay (for compound screening). Forty microliter CellTiter-Blue® Reagent (Promega, G8081) was added to 200 μL culture medium at a 96-well plate (or 20% volume of the total medium). The plate was then shaken for 10 s and incubated in a cell culture incubator for 3 h. The fluorescence of optical density (O.D.) 560/590 was measured by a spectrophotometer (BioTek, VT, USA), with the baseline ratio (measured in no-cell control wells) subtracted.

### Immunofluorescence staining

For immunofluorescence staining, cells were first fixed in 4% PFA at room temperature for 15 minutes. After washing with PBS, the cells were permeabilized with 0.1% Triton X-100 in PBS for 10 min at room temperature. To block non-specific binding, the cells were incubated with 5% BSA in PBS for 1 hour at room temperature. Following blocking, the cells were incubated with primary antibodies diluted in 1% BSA in PBS overnight at 4 °C. The next day, the cells were washed three times with PBS and incubated with appropriate fluorophore-conjugated secondary antibodies for 1 h at room temperature in the dark. After washing the cells three times with PBS, nuclei were counterstained with DAPI (1:2000 dilution) for 5 min. The coverslips were then mounted onto glass slides using an anti-fade mounting medium, and images were acquired using an A1R laser confocal microscopy (Nikon, Japan).

### Colony-forming assay

A total of 100–300 UCB-derived CD34^+^ cells or 5 × 10^4^–2 × 10^5^ PBMCs were resuspended in 1–2 mL methylcellulose medium (StemCell Technologies, Methocult H4435 or Methocult H4330 EPO only) containing 1–2 μL ciprofloxacin and 1–2 μL compounds dissolved in DMSO at the required concentrations and incubated at 4 °C for 10 min. Methocult H4435 was used unless noted otherwise. The cells were seeded into a 6-well or 12-well plate using a 1-mL blunt-ended syringe, and PBS was added to the spaces between the wells to avoid drying out. After the 14-day incubation, plates were photographed under a microscope, following which PBS was added for elution, counting, and flow cytometry, if necessary. The colony area was measured using ImageJ v. 1.53q. Except where specifically mentioned: SB, 1 μM SB-590885; GDC, 2 μM GDC-0879; Enco, 500 nM Encorafenib, and these compounds were only added once at the beginning of seeding.

## Supplementary information

Supplemental material

## Data Availability

High-throughput sequencing data are available in the NCBI Gene Expression Omnibus database GSE221242, GSE261119 and GSE275226. The mass spectrometry proteomics data have been deposited to the ProteomeXchange Consortium with the dataset ID PXD055730.
