# Supplementary material for: BRAF inhibitors enhance erythropoiesis and treat anemia through paradoxical activation of MAPK signaling
Source: Signal Transduct Target Ther. 2024 Dec 2;9:338. doi: 10.1038/s41392-024-02033-6 (PMC11609275; doi:10.1038/s41392-024-02033-6)
Supplement: Supplementary file 1 — Supplemental material [file 41392_2024_2033_MOESM1_ESM.docx]

Supplementary Materials for

**BRAF inhibitors enhance erythropoiesis and treat anemia through paradoxical activation of MAPK signaling**

Shunkang Wu, Yuelin Deng, Haobo Sun, Xuewen Liu, Shuo Zhou, Hanxi Zhao, Huan Li, Fusheng Guo, Qiuyu Yue, Fan Wu, Xinying Zhao, Na Li, Shicong Zhu, Qi Hu, Si Xie, Jie Zheng, Meng Lv, Yuan Kong, Xiao-Jun Huang, Xiaoguang Lei, Xiangmin Tong, Xiaofei Gao, and Hsiang-Ying Lee

Correspondence to: Hsiang-Ying Lee, slee@pku.edu.cn; Xiaofei Gao, gaoxiaofei@westlake.edu.cn; Xiangmin Tong, tongxiangmin@163.com

**This PDF file includes:**

Materials and Methods

Supplementary References

Figures. S1 to S9

Tables S1 to S4

Materials and Methods

**Cell isolation**

Umbilical cord blood derived-human CD34^+^ cells (UCB-derived CD34^+^ cells): Human hematopoietic stem/progenitor cells (HSPCs, UCB-CD34^+^ cells) were purified from cord blood samples obtained from the Cord Blood Bank of Beijing.

Peripheral blood mononuclear cells (PBMCs): Human peripheral blood mononuclear cells were purified from peripheral blood samples provided by Peking University People's Hospital. The DBA patients’ peripheral blood samples were provided by Beijing Children's Hospital affiliated with Capital Medical University.

All mononuclear cells (MNCs) were isolated by density gradient centrifugation no longer than 24 hours after sample collection. MNCs were mixed with diluted human FcR blocking reagent, and UCB-derived CD34^+^ cells were isolated using a MACS MicroBead kit (Miltenyi, 130-100-453, GMBH). Flow cytometry was used to assess the purity of isolated CD34^+^ cells, and all CD34^+^ cells used in this study were > 90% CD34 positive.

**The primary human erythroid and HSPCs culture system**

UCB-derived CD34^+^ cells or MNCs were cultured in a modified 14-day erythroid differentiation system reported previously^S1^. For screening, 200 UCB-derived CD34^+^ cells were seeded in 200 μL erythroid culture medium and cultured in a 96-well plate for 7 days. Cells were treated with different concentrations of compounds in DMSO, or DMSO as a control, at no higher than 0.1% v/v of the total medium. GDC-0879 (MCE, HY-50864), SB-590885 (MCE, HY-10966) and Encorafenib (MCE, HY-15605) were dissolved in DMSO for *in vitro* culture.

In general, in 14-day erythroid culture system and HSPCs retention culture system, except where specifically mentioned: SB, 1 μM SB-590885; GDC, 2 μM GDC-0879; Enco, 500 nM Encorafenib. Medium and compounds were refreshed after centrifugation every 3 days, and compound administration ceased on day 12 as described in **Fig. 1a**. The compounds used for screening were selected from the MedChemExpress (MCE) FDA-Approved Drug Library (MCE, HY-L022).

For CD34^+^ HSPCs retention culture: HSPCs were cultured in SFEM II (StemCell Technologies, 09605), supplemented with 100 ng/mL human SCF (StemCell Technologies, 78062), 100 ng/mL human FLT3L (StemCell Technologies, 78137), 100 ng/mL human TPO (StemCell Technologies, 78210), 500 nM SR1 (MCE, HY-15001) and 35 nM UM171 (MCE, HY-12878). The medium was replaced halfway every other day, and the cell density was maintained at less than 1 million cells per mL.

For cytokine-restricted experiments, the “5% EPO” condition refers to the regular erythroid culture medium, except with 5% of the regular amount of EPO (0.15 IU/mL EPO); “0% SCF” condition refers to the regular erythroid culture medium, except without SCF. Except for the indicated cytokine, the concentrations of other cytokines were kept unchanged as described in the paragraph above.

**The primary human erythroid and HSPCs culture system**

UCB-derived CD34^+^ cells or MNCs were cultured in a modified 14-day erythroid differentiation system reported previously^S1^. For screening, 200 UCB-derived CD34^+^ cells were seeded in 200 μL erythroid culture medium and cultured in a 96-well plate for 7 days.

**Cell apoptosis assay**

Human CD34^+^ cells were cultured and treated for 5 days in erythroid differentiation medium, then transferred to fresh cytokine-free medium. Cells were resuspended in Annexin V binding buffer (Invitrogen, BMS500BB) and stained with Annexin V-FITC according to the manufacturer’s instructions. Propidium iodide (PI) was added to the suspension at 100 ng/mL prior to flow cytometry analysis.

**Benzidine-Giemsa staining**

A total of 1 × 10^5^ erythrocytes were resuspended in 200 μL PBS, fixed in methanol at -20°C, and concentrated on a slide by cytocentrifugation using cytospin (Thermo, MA, USA) at 200 rpm for 5 min. The slide was washed with distilled water and air-dried. One benzidine tablet (Sigma, D5909) was dissolved in 10 mL PBS, 10 μL 20% H_2_O_2_ was added, and the solution was passed through a 0.22-μm filter membrane. The cytospin slide was incubated with 200–500 μL benzidine solution for 1 h at room temperature, after which the slide was incubated with Giemsa staining solution diluted 1:20 in distilled water at room temperature for 35–45 min. Cells were washed with distilled water, air-dried, and covered with coverslips for observation under a microscope.

**Multicolor flow cytometry**

The antibodies list used for flow cytometry can be found in **supplemental table 1**. In brief, cells at the specified time point were resuspended and filtered for counting and cell density assay. Cells were resuspended in PBS at approximately 1 × 10^6^ per mL and incubated at room temperature for 10 min with mouse IgG at 1:100, followed by incubation with antibodies for specific cell surface markers (anti-CD34, anti-CD117, anti-CD45RA, anti-CD38, anti-CD90, anti-CD14, anti-CD11c, anti-CD11b, anti-CD235a, anti-CD71 were stained at 1:100), and 10 μg/mL Hoechst33342 (Invitrogen, R37165) or DAPI (Invitrogen, 62248) at 1:2000, or 7-AAD (Invitrogen, 00-6993-50) at 1:200. When staining with Hoechst33342, specific antibodies and Hoechst33342 were incubated together at 37°C for 30 min.

For the detection of intracellular markers, cells were first fixed by resuspension in 2% BSA and incubation with 4% paraformaldehyde (PFA) at room temperature for 10 minutes. Following fixation, the cells were permeabilized by resuspending in 0.1% Triton X-100 in 2% BSA and incubating at room temperature for 15 minutes. Then the cells were incubated with the primary antibody, rabbit anti-pERK (CST, 4370), at a 1:1000 dilution at 4°C for 1 hour in 2% BSA. The cells were then washed and incubated with the goat anti rabbit IgG-FITC secondary antibody (Invitrogen, 31573) along with the necessary surface marker flow cytometric antibodies, at a 1:200 dilution at 4°C for 1 hour in 2% BSA. For the negative control, no primary antibody was added, but the secondary antibody and surface flow cytometry antibodies were added as usual.

For the detection of mouse erythroid lineage in bone marrow cells, the cells were resuspended in 2% BSA and incubated with Mouse BD Fc Block™ Purified Rat anti-CD16/CD32 (BD, 553142), anti-CD45R(B220) (BD, 51-01122J), anti-CD3e (BD, 51-01082J), anti-GR1 (BD, 51-01212J), and anti-CD11b (BD, 51-01712J) biotinylated-antibody at 4°C at approximately 1×10^7^ cells per mL at a 1:100 dilution for 1h. Subsequently, the cells were incubated with Streptavidin-BV711 (BioLegend, 405241), CD71-PE (BioLegend, 113808), and TER119-FITC (Invitrogen, 2056566) at a 1:200 dilution at 4°C for 1h in 2% BSA. DAPI was added at a 1:2000 dilution before the analysis.

For the detection of human engraftment in mouse peripheral blood, we first lysed the whole blood erythrocytes using a red blood cell lysis solution (Beyotime, C3702) as the provided instructions. Then cells were resuspended in 2% BSA and incubated with human FcR Blocking Reagent (Miltenyi, 130-059-901), anti-mouse CD45 (BD, 557659), anti-human CD45 (BD, 563879), anti-hCD19 (Invitrogen, 25-0198-41), anti-hCD3 (Invitrogen, 56-0038-80), anti-hCD33 (Invitrogen, 12-0339-42), and anti-hCD14 (Invitrogen, 11-0149-42) at 4℃ at approximately 1×10^7^ cells per mL at a 1:100 dilution for 1h. Before analysis, 7-AAD was added at a 1:300 dilution. The use of other flow cytometry antibody combinations is as mentioned in the text.

Flow cytometry analysis was performed using an LSRFortessa™ (BD, NJ, USA). For sorting of specifically labeled cell populations, a FACSAria™ lll (BD, NJ, USA) was used. Flow cytometry data were processed using FlowJo v10.5.3.

**Immunoblotting**

Prior to collection, 1 × 10^6^ per mL cells were incubated for 30 min with fresh medium containing the appropriate compounds (No any chemicals treatment before). Then cells were collected and washed in PBS by centrifugation. Cell pellets were resuspended in 200 μL diluted SDS loading buffer and boiled at 100°C for 15 min. The antibodies used for immunoblotting are listed in **supplemental table 2**. Immunoblotting was performed according to standard procedures^S2^.

For some cases, after the initial detection, the membranes were stripped using a stripping buffer (Beyotime, P0025) at room temperature for 30 minutes with gentle shaking. The membranes were then washed extensively in TBST and blocked again with 5% non-fat milk in TBST for 1 hour at room temperature. Following blocking, the membranes were re-incubated with the appropriate primary antibodies, and the detection procedure was repeated as per standard protocols.

**Plasmid transfection and lentiviral transduction**

HEK293T cells were seeded one day prior to transfection and cultured to 60–70% confluence, after which the fresh antibiotic-free medium was added. A total of 7.5 μg plasmid DNA and 3.75 μg lentiviral packaging plasmids pVSV-G (Addgene, 35616) and psPAX2 (Addgene, 12260) were added to serum-free DMEM, mixed with DMEM containing Lipo293 liposomes (Beyotime, C0521), and incubated at room temperature for 30 min. Subsequently, the transfection solution was added dropwise to cells and incubated for 48 hr. After 48 hr. of transfection, the medium was passed through a 0.22-μm filter membrane, 5× PEG 8000 was added, and the medium was mixed well and incubated at 4°C overnight. The next day, the medium was centrifuged at 3500 × g for 40 min, the supernatant was discarded, and the lentivirus concentrate was obtained by resuspending the precipitated virus in 100 μL PBS. A total of 1 × 10^5^ cells were resuspended in 200 μL antibiotic-free medium and 100× Polybrene was added. After centrifugation at 300 × g for 120 min, cells were seeded and cultured, and the medium was replenished to 500 μL 8 hr post-transduction. Two days later, GFP intensity indicating transduction efficiency was measured by flow cytometry, and GFP-based sorting was performed as described previously^S3^. The pMSCV-GFP vehicle vector or lentivirus was transferred into both the Ctrl group and the GDC-treated group. The shRNA sequences are listed in **supplemental table 3**.

**Quantitative real-time PCR**

Total RNA from 5x10^6^ cells were extracted using the RNeasy Mini Kit (Qiagen, 74104) according to the manufacturer's instructions. Subsequently, 1 μg of RNA was reverse-transcribed into cDNA using the PrimeScript™ RT reagent Kit (Takara, RR037). Quantitative real-time PCR (qPCR) was conducted with TB Green^®^ *Premix Ex Taq*^™^ II system (Takara, RR802) on LightCycler 96 system (Roche, Swiss). The thermal cycling conditions were as follows: initial denaturation at 95°C for 30 seconds, followed by 40 cycles of 95°C for 5 seconds and 60°C for 30 seconds. The *Cq* values for target gene were normalized to *Gapdh*, and the relative expression changes were calculated using the ΔΔ*Cq* method. The primers used for target gene in qPCR are listed in **supplemental table 4**.

**RNA extraction and transcriptome sequencing/analysis**

Approximately 1 × 10^6^ cells were used for RNA extraction. Cells were washed once with 1–2 mL pre-chilled PBS, resuspended in 1 mL TRIzol (Vazyme, R401-01), and incubated for 5 min at room temperature. Subsequently, 250 µL chloroform was added to the cell suspension, shaken vigorously for 15 s, and incubated for 5 min at room temperature. The cell suspension was centrifuged at 12,000 rpm for 15 min at 4°C, the aqueous phase was transferred to a clean 1.5-mL EP tube, 550 µL isopropanol was added and mixed gently, and the solution was incubated at room temperature for 5 min. The solution was centrifuged again at 4°C for 10 min at 12,000 rpm, the supernatant was discarded, and the RNA precipitate was resuspended in 1 mL pre-chilled 75% ethanol. The solution was centrifuged again at 7,500 rpm for 5 min, the supernatant was discarded, and the RNA precipitate was air-dried and resuspended in approximately 15-25 µL TE buffer or RNA-free water. The absorbance was measured at 260 nm and 280 nm to confirm that the 260/280 ratio was greater than 1.8 and the concentration was higher than 50 ng/µL.

Total RNA quality was assessed using Agilent 2100 bioanalyzer and then 1 μg of total RNA was quantified using the Qubit High-sensitivity kit (Life Technologies, Q32852), and the RNA-seq library was constructed by NEBNext Ultra^TM^ RNA Library Prep Kit for Illumina (NEB, E7530L). Two biological replicates per treatment were sequenced in a Novaseq 6000 (Illumina, PE150) by Novogene Co. (Beijing)^S4^.

The raw data of RNA-seq was first quality checked using FastQC (https://www.bioinformatics.babraham.ac.uk/projects/fastqc/). Hisat2^S5^ was used to align the paired-end reads to hg19 version of human reference genome and estimate the reads counts with HTSeq^S6^. The commands were: “hisat2 -x hg19 -t -p 8 -1 fq1 -2 fq2 -S sample.sam; htseq-count -f bam sample.bam”. We used samtools^S7^ to covert files format and generate index files. The read counts were normalized to TPM (transcription per million)^S8^, which is a measurement of RNA transcripts.

Differentially expressed genes (DEGs), Coexpression atlas analysis, and the Molecular Signatures Database (MSigDB) hallmark gene sets enrichment analyses were performed on the online integrated website software iDEP v0.96/v1.0^S9, 10^. Gene Ontology and KEGG enrichment analysis were performed on the graphic user interface (GUI)-based visualization application Hiplot (https://hiplot.com.cn/)^S11^, GENE ONTOLOGY (GO) TOOLS (https://go.princeton.edu/) or using Toppgene (https://toppgene.cchmc.org/)^S12^. GOBP with visualization was plotted with Revigo (http://revigo.irb.hr/)^S13^.

**ATAC-seq library construction and sequencing/analysis**

In brief, 10^3^ transduced or treated day 5 maintained CD34^+^ cells or 10-day differentiated electroporated erythroid cells from UCB-CD34^+^ or were lysed on ice for 5 min in lysis buffer (10 mM Tris-HCl (pH 7.4), 10 mM NaCl, 3 8 mM MgCl_2_ and NP-40) to extract the nuclei. Post lysis, cells were centrifuged at 500 g for 5 min, discarding the supernatant. The nuclei were then incubated with Tn5 transposase and the corresponding buffer at 37°C for 30 min (Vazyme, TD501). The stop buffer was added to the reaction to terminate the tagmentation. DNA Clean Beads were used to purify DNA. PCR was subsequently applied to append indices to the fragments. Two biological replicates for each sample were sequenced on a Novaseq 6000 (PE150, Illumina, CA, USA) by Novogene (Beijing). ATAC-seq was performed as previously described^S14^.

The adaptors were removed from the raw ATAC-seq paired data using trim_galore software with default parameters. Trimmed cleaned reads were aligned to the human reference genome hg19 with Bowtie2 (version 2.3.4.1)^S15^ with default parameters and converted to bam files using samtools^S7^. ATAC-seq peaks were identified using MACS2^S16^ with the following parameters: “-q 0.05 --nomodel --shift -100 --extsize 200”. Signal enrichment plots around the TSS of all protein-coding genes was generated using deeptools. The example tracks were visualized using IGV software^S17^. Motif enrichment in ATAC-seq peaks was conducted using Homer software^S18^. The foldchange of cutoff of the difference peak is taken as log_2_0.2.

**Affinity immunoprecipitation-mass spectrometry**

10^7^ Cells overexpressed 3×Flag-BRAF^WT^ or 3×Flag-BRAF^V600E^ were lysed in 1 mL of NP-40 lysis buffer (Beyotime, P0013F) containing protease inhibitors (MCE, HY-K0010) on ice. The lysate was incubated overnight at 4°C with 20 μL of anti-Flag magnetic beads (MCE, HY-K0270) that had been pre-washed three times with wash buffer (50 mM Tris-HCl, 150 mM NaCl, 0.5% Tween-20, pH 7.4). Following incubation, the magnetic beads were separated using a magnetic stand and washed three additional times with wash buffer. The bound proteins were then eluted by adding 50 μL of 1× SDS-PAGE loading buffer, followed by heating at 95°C for 5 minutes. The supernatant was collected after magnetic separation and subjected to SDS-PAGE. The gel bands of interest were excised and subjected to in-gel digestion. The gel pieces were first destained with 400 μL of destaining solution, then dehydrated with 400 μL of acetonitrile until the gel pieces turned white. The gel pieces were reduced with 200 μL of 10 mM DTT/25 mM NH_4_HCO_3_ at 56°C for 1 hour and then alkylated with 200 μL of 55 mM IAA/25 mM NH_4_HCO_3_ for 45 minutes in the dark. After washing twice with 25 mM NH_4_HCO_3_ and dehydrating with acetonitrile, the gel pieces were digested overnight at 37°C with trypsin in 25 mM NH_4_HCO_3_ at a 1:50 enzyme-to-protein ratio. Peptides were extracted twice with 200 μL of acetonitrile containing 0.1% formic acid, and the supernatant was combined and vacuum-dried. The dried peptides were reconstituted in 0.1% formic acid for LC-MS/MS analysis. The samples were loaded onto a C18 column and analyzed using an EASY-nLC 1200 system (Thermo, MA, USA) coupled to a Thermo Orbitrap Exploris 480 (Thermo, MA, USA) mass spectrometer. The mobile phase consisted of 0.1% formic acid (A) and 80% acetonitrile/0.1% formic acid (B), with a flow rate of 300 nL/min. The mass spectrometry data were processed using Proteome Discoverer 2.2 software against the UniProt Homo sapiens (Human) database.

The protein abundance and ratio of each group in IP-MS were calculated by Proteome Discoverer. After obtaining the protein abundance of each group, the values ​​were scaled, and then calculated and processed by Deseq2^S19^ to obtain differentially expressed proteins, and then the volcano map was drawn using hiplot^S11^. For the most significantly up-regulated and down-regulated proteins between each group, protein abundance was firstly scaled and the differentially expressed proteins were then processed by limma^S20^ package and a heatmap was drawn. To generate a heatmap containing three groups of samples, the top 50 proteins with the most significant differences were extracted, and the significance of all proteins in the three groups of samples was calculated using ANOVA analysis, and then the top 50 significant proteins were extracted and a heatmap was generated.

***In vivo* mouse experiments**

All mice were fed regular chow and housed in a specific pathogen-free animal facility under a 12/12 h light/dark cycle at 22°C at the Beijing Vital River Laboratory, Westlake University, innomodel inc. Biotechnology (Beijing) or Peking University.

Routine blood examination in mice: 30 μL of blood was collected from the eye socket or tail vein into a K_2_-EDTA tube. The blood was examined using a fully automated hematology analyzer BC5000 (Myriad, PRC).

*In vivo* mouse experiments (*Rpl11* mice generation): Generation of a conditional *Rpl11* haploinsufficiency mouse model: A donor DNA vector containing the entire *Rpl11* gene flanked by loxP sites were constructed. The sgRNA, Cas9 and donor DNA were microinjected into the fertilized eggs from C57BL/6JGpt mice. The fertilized eggs were then transplanted into pseudo-pregnant C57BL/6JGpt female mice to yield F0 mice. Positive F0 mice were confirmed by PCR genotyping to verify the presence of loxP sites and subsequently bred with wild type C57BL/6JGpt mice to yield F1 progeny for germline transmission verification. *Rpl11*^+/lox^ F1 mice were crossbred with mice expressing inducible Cre (Tg.hUbC-CreERT2). The mice used in this work are of B6 background. All *Rpl11* mice were housed at Laboratory Animal Resources Center of Westlake University under pathogen-free conditions. Mice were fed with standard chow diet ad libitum. The procedures were approved by the Ethical Committee of Westlake University.

*Rpl11* haploinsufficiency mouse model: For *Rpl11* conditional haplo-knockout, 6- to 8-week-old male *Rpl11*^+/lox^ mice containing CreERT2 were intraperitoneally injected with tamoxifen dissolved in corn oil (20 mg/mL) at approximately 75 µL (75 mg/kg body weight) once daily for five consecutive days. Following the final injection, mice were quarantined for 24 hours before returning to standard housing. Once the mice developed sustained anemia, they received daily oral gavage treatments of GDC-0879 (50 mg/kg) or a vehicle starting from the seventh week for a period of 5 weeks.

Cisplatin-induced anemia model (**Fig. 6g**): Cisplatin was administered intraperitoneally (I.P.) to 6- to 8-week-old C57BL/6N mice at 7 mg/kg once a week for 2 weeks, followed by 3.5 mg/kg I.P. once a week for the subsequent 2 weeks. After a week of acclimatization, mice were randomly divided into 2 groups based on weight. The mice were administrated 30 mg/kg GDC-0879 or vehicle via oral gavage for 3 weeks. All mice were euthanized at the end of the treatment period.

Cisplatin-induced anemia model (**supplementary fig. 9g**): 6- to 8-week-old C57BL/6N mice were randomly divided into 2 groups based on weight and cisplatin was administered I.P. at 7 mg/kg once a week for 2 weeks. At the same time, mice were administrated 30 mg/kg GDC-0879 or vehicle via oral gavage once daily for 2 weeks. All mice were euthanized at the end of the treatment period.

**Transplantation in mice and assessment of human cell chimerism**

Human CD34^+^ cell transplantation in NPSG mice:

Four-week-old female NOD.Cg-*Prkdc^scid^ Il2rg^tm1Wjl^*/Pntk mice (InnoModels Biotechnology, Beijing) were irradiated (160 rads) 24 hours prior to transplantation. The next day, the mice were briefly sedated with isoflurane, and then received a transplant of about 8 x 10^4^ human cord blood CD34^+^ cells in a 200 μL volume of HBSS via a 29.5-gauge insulin needle. After 10 weeks, the mice were randomly divided into 2 groups, and treatment with GDC-0879 (20 mg/kg) or a vehicle was initiated, administered via oral gavage every other day from week 10 for a total of 4 weeks.

Human CD34^+^ cell transplantation in NCG-X mice:

Ten-week-old female triple-immunodeficient NCG mouse with a point mutation in *Kit* (CD117) NCG-X mice (NOD/ShiLtJGpt-*Prkdc^em26Cd52^Il2rg^em26Cd22^kit^em1Cin(V831M)^*/GptCrl) were purchased from GemPharmatech Co., Ltd. Before transplantation, the mice were briefly sedated with isoflurane. We then transplanted approximately 8 x 10^4^ human cord blood CD34^+^ cells in a 200 μL volume of HBSS using a 29.5-gauge insulin needle. After 4 weeks of care, the mice were randomly divided into 2 groups. Starting from week 4 and continuing for 4 weeks, GDC-0879 (20 mg/kg) or a vehicle was administered every other day via oral gavage.

For NCG-X and NPSG mice, we collected 100 μL of blood from the tail vein weekly during the dosing period to assess the chimerism of human leukocyte engraftment in the peripheral blood, using previously described analysis procedures^S21^.

After four weeks of dosing, the mice were euthanized. The bone marrow and spleen of mice were processed into cell suspensions in PBS containing 2% FBS, amounting to 10 mL for bone marrow and 5mL for spleen. To preserve human red blood cells, samples did not undergo erythrocyte lysis. The cell suspensions were stained with the following flow cytometric antibody panels to determine the chimerism ratio of the human hematopoietic system.

For bone marrow lineage: mouse: CD45 APC-Cy7 (BD, 557659); human: CD45 BV421(BD, 563879), CD19 PE-Cy7 (Invitrogen, 25-0198-41), CD3 AF700 (Invitrogen, 56-0038-80), CD33 PE (Invitrogen, 12-0339-42), CD14 FITC (Invitrogen, 11-0149-41), and CD11b APC (Invitrogen, 17-0118-41).

For bone marrow erythroid and megakaryocyte lineage: mouse: CD45 APC-CY7 (BD, 557659), Ter119 FITC (Invitrogen, 2056566); human: CD45 BV421(BD, 563879), CD41 AF700 (Invitrogen, MA5-28562), CD71 PE (Invitrogen, 12-0719-42), and CD235a APC (Invitrogen, 17-9987-42).

For bone marrow hematopoietic progenitor cells: mouse: CD45 APC-CY7 (BD, 557659), Ter119 FITC (Invitrogen, 2056566); human: CD45 BV421(BD, 563879), CD34 AF700 (BD, 561440), CD38 BV605 (BD, 562665), CD45RA BV711 (BD, 563733), and CD90 PE-CY7 (BD, 561558). Each antibody was used at a 1:100 dilution. Prior to flow cytometry analysis, all samples were stained with 7-AAD at a 1:500 dilution. Compensation for the staining was achieved using automated compensation calculation using UltraComp beads from Invitrogen. The BD Fortessa cytometer was used for acquisition, and FACS Aria III cell sorter was utilized for sorting purposes.

**Statistical analysis and graph**

Statistical analysis was performed using GraphPad Prism 9.3.1. Data are expressed as the mean ± standard deviation. A two-tailed unpaired/paired Student's *t*-test was performed for the comparison between two groups. A one-way ANOVA was performed in existing more than 2 groups’ comparison. A two-way ANOVA was performed to analyze the effects of time and treatment as variables. (ns, *P*＞0.05; *, *P* < 0.05; **, *P* < 0.01; ***, *P* < 0.001; ****, *P* <0.0001).

Heatmap, bubble plot, tree graphs and connectivity map were created using the graphic user interface (GUI)-based visualization application Hiplot^S11^ and GraphPad Prism 9.

**Supplemental References**

S1. Lee, H. Y. et al. PPAR-alpha and glucocorticoid receptor synergize to promote erythroid progenitor self-renewal. *Nature* **522**, 474-477 (2015).

S2. Litovchick L. Immunoblotting. *Cold Spring Harb Protoc*. **2020(6)**, pdb-top098392 (2020)

S3. Cormack, B.P., Valdivia, R.H. & Falkow, S. FACS-optimized mutants of the green fluorescent protein (GFP). Gene **173**, 33-38 (1996).

S4. Li, D., Wu, F., Zhou, S., Huang, X.J. & Lee, H.Y. Heterochromatin rewiring and domain disruption-mediated chromatin compaction during erythropoiesis. Nat. Struct. Mol. Biol. **30**, 463-474 (2023).

S5. Kim, D., Langmead, B. & Salzberg, S.L. HISAT: a fast spliced aligner with low memory requirements. Nat. Methods **12**, 357-360 (2015).

S6. Anders, S., Pyl, P.T. & Huber, W. HTSeq--a Python framework to work with high-throughput sequencing data. Bioinformatics **31**, 166-169 (2015).

S7. Li, H., Handsaker, B., Wysoker, A., Fennell, T., Ruan, J., Homer, N., et al. The Sequence Alignment/Map format and SAMtools. Bioinformatics **25**, 2078-2079 (2009).

S8. Wagner, G.P., Kin, K. & Lynch, V.J. Measurement of mRNA abundance using RNA-seq data: RPKM measure is inconsistent among samples. Theory Biosci. **131**, 281-285 (2012).

S9. Ge, X. Idep Web Application for RNA-Seq Data Analysis. Methods Mol. Biol. **2284**, 417-443 (2021).

S10. Ge, S.X., Son, E.W. & Yao, R. iDEP: an integrated web application for differential expression and pathway analysis of RNA-Seq data. BMC Bioinformatics **19**, 534 (2018).

S11. Li, J. et al. Hiplot: a comprehensive and easy-to-use web service for boosting publication-ready biomedical data visualization. Brief Bioinform. **23**, (2022).

S12. Chen, J., Bardes, E.E., Aronow, B.J. & Jegga, A.G. ToppGene Suite for gene list enrichment analysis and candidate gene prioritization. Nucleic Acids Res. **37**, W305-W311 (2009).

S13. Supek, F., Bosnjak, M., Skunca, N. & Smuc, T. REVIGO Summarizes and Visualizes Long Lists of Gene Ontology Terms. PLoS One **6**, (2011).

S14. Wu, J. et al. The landscape of accessible chromatin in mammalian preimplantation embryos. Nature **534**, 652-657 (2016).

S15. Langmead, B. & Salzberg, S.L. Fast gapped-read alignment with Bowtie 2. Nat. Methods **9**, 357-359 (2012).

S16. Zhang, Y. et al. Model-based analysis of ChIP-Seq (MACS). Genome Biol. **9**, R137 (2008).

S17. Robinson, J.T. et al. Integrative genomics viewer. Nat. Biotechnol. **29**, 24-26 (2011).

S18. Heinz, S. et al. Simple combinations of lineage-determining transcription factors prime cis-regulatory elements required for macrophage and B cell identities. Mol. Cell **38**, 576-589 (2010).

S19. Love, M.I., Huber, W. & Anders, S. Moderated estimation of fold change and dispersion for RNA-seq data with DESeq2. *Genome Biol* **15**, 1-21 (2014).

S20. Ritchie, M.E., Phipson, B., Wu, D.I. *et al.* limma powers differential expression analyses for RNA-sequencing and microarray studies. *Nucleic Acids Res* **43**, e47 (2015).

S21. Doulatov, S. et al. Drug discovery for Diamond-Blackfan anemia using reprogrammed hematopoietic progenitors. Sci. Transl. Med. **9**, (2017).

Figure. S1.


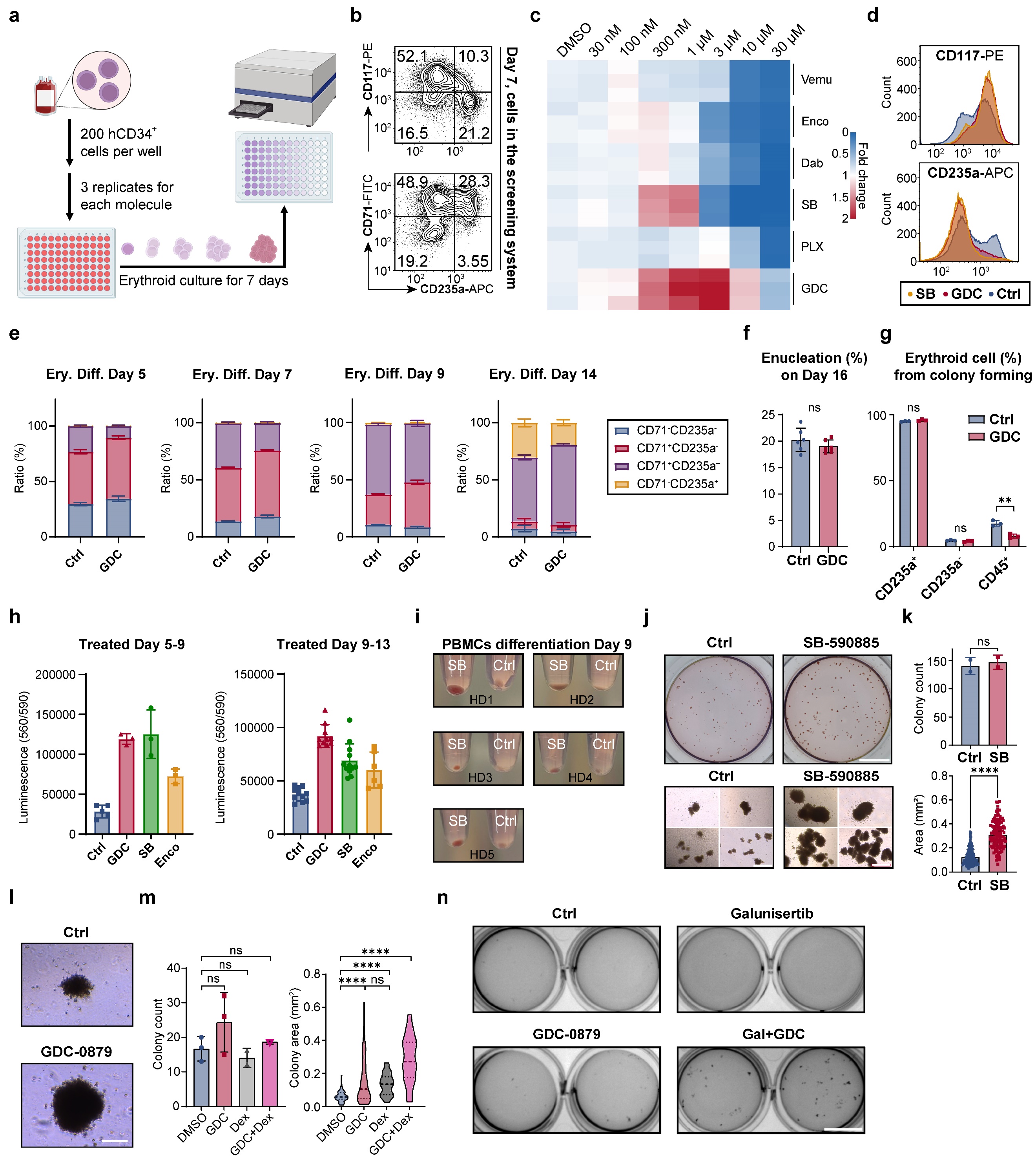


**Figure. S1. BRAF inhibitors expanded erythroid progenitors from UCB-CD34^+^ cells and PBMCs and delayed differentiation *in vitro*.**

**a** Schematic abstract of a 7-day pro-proliferative compound screening strategy for human primary erythroid cells. Compounds were only added once at the beginning of the culture of 200 UCB-CD34^+^ cells and measured cell number using CellTiter-Blue® Reagent by luminescence on Day 7. **b** FACS analysis showing typical erythroid surface marker expression of control cells on Day 7 in the 96-well plate screening system in panel **a**. **c** Fold change in cell number measured on Day 7 in a 96-well plate with different concentrations of BRAFi (30 nM to 30 μM) or DMSO control. n = 3 for each compound and concentration. **d** Representative differences in erythroid surface markers between control (DMSO), SB-treated, and GDC-treated cells on Day 7 in the screening system using a 96-well plate. **e** Quantification of the proportions of erythroid cells at differentiation Day 5, 7, 9 and 14 (from a separated batch than **Fig. 1d**). n = 3. **f** Enucleation rate (determined by the CD235a^+^Hoechst33342^-^ population (as shown in **Fig. 1d**) between control and 2 μM GDC-treated cells (only treated from Day 0 to 9) and enucleation ratio were calculated on Day 16. n = 6. **g** Quantification of erythroid and other myeloid cell ratio recovered from the colonies in erythroid progenitor cells colony forming assays (CFA) in **Fig. 1j**. n = 3. **h** Luminescence assay measuring cell proliferation from Day 5 to Day 9 (Left) and Day 9 to Day 13 (Right). CD34^+^ cells were treated with different compounds. GDC 2 μM, SB 1 μM, Enco 500 nM. **i** Cell pellets of Day 9 erythroblasts derived from 0.5 million PBMCs from Day 0. **j** (Top) Whole-plate view of CFA with 2 × 10^5^ PBMCs from healthy donors seeded in Methocult H4435 and incubated under SB-treated or control conditions for 14 days. Scale bar = 10 mm. (Bottom) Representative images of large BFU-E colony clusters and single colonies. Scale bar = 500 μm. **k** Statistical analysis of erythroid colony count and colony area in panel **j**. **l** and **m** Representative erythroid colony picture of 5×10^4^ health donor PBMCs CFA in methocult H4435 for 14 days (panel **l**). Erythroid colony count and erythroid colony area statistics on Day 14 (panel **m**). **n** Whole-plate view of colony forming assays (CFA) with 100 UCB-CD34^+^ cells seeded in EPO-only methylcellulose medium H4430, treated with 2 μM GDC, 500 nM TGF-β inhibitor (TGFβi) Galunisertib, and GDC with Galunisertib. The photo was taken on Day 14. Scale bar = 10 mm. GDC, 2 μM GDC; Dex, dexamethasone 0.1 μM. Vemu, Vemurafenib; Enco, Encorafenib; Dab, Darbrafenib; SB, SB-590885; PLX, PLX8394; GDC, GDC-0879. Unless otherwise specified, the following concentrations of BRAFi were used: 1 μM SB-590885 (SB), 2 μM GDC-0879 (GDC), and 500 nM Encorafenib (Enco). The control group (Ctrl) was treated with DMSO. Data are shown as the mean ± SD. An unpaired Student’s *t*-test for the comparison between two groups. ns, not significant, **, *P* < 0.01; ****, *P* < 0.0001.

Figure. S2.


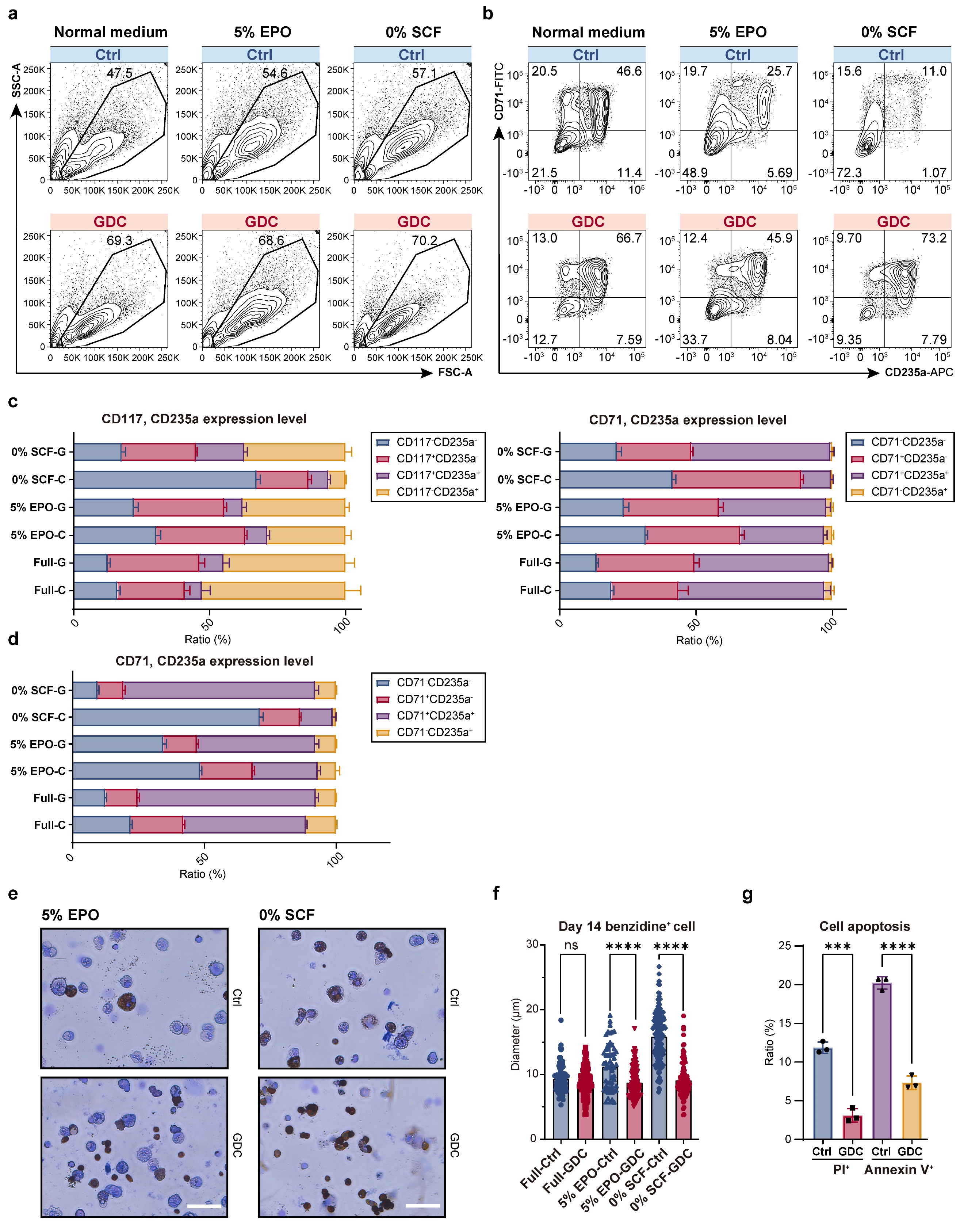


**Figure. S2. BRAF inhibitors improved erythroid development under cytokine-restricted conditions *in vitro*.**

**a** and **b** Flow cytometry analysis of UCB-CD34^+^-derived erythroid cells in the control group and GDC-0879-treated group on Day 14 of differentiation under normal and specified conditions: 5% EPO (0.15 IU/mL EPO); 0% SCF (0 ng/mL SCF) using SSC-A and FSC-A, and surface markers CD71 and CD235a. Concentrations of other cytokines were kept unchanged as usual. **c** Quantification of the proportions of erythroid cells on Day 9 of differentiation using surface markers CD117, CD71 and CD235a. **d** Quantification of the proportions of erythroid cells on Day 14 of differentiation using surface markers CD71 and CD235a. **e**. Representative micrographs of Benzidine-Giemsa staining for UCB-CD34^+^ derived erythroid cells cultured under different cytokine-restricted conditions on differentiation Day 14. Scale bar = 50 μm. **f** Statistical analysis of the diameters of benzidine-positive cells measured under a microscope after 14 days of differentiation, as shown for each condition in panel **e**. Full-Ctrl, n = 81; Full-GDC, n = 155, 5%-EPO-Ctrl, n = 56; 5%-EPO-GDC, n = 102; 0%-SCF-Ctrl, n = 141; 0%-SCF-GDC, n = 111. **g** Quantification of the apoptotic cell ratio after 48h cytokine deprivation in Day 5 erythroid cells, as shown in **Fig. 2f.** Error bars represent the mean ± SD. n = 3 in panel **c**, **d** and **g**. An unpaired two-tailed Student's *t*-test was performed for the statistical comparison between two groups (ns, *P*＞0.05; ***, *P* < 0.001; ****, *P* < 0.0001).

Figure. S3.

**
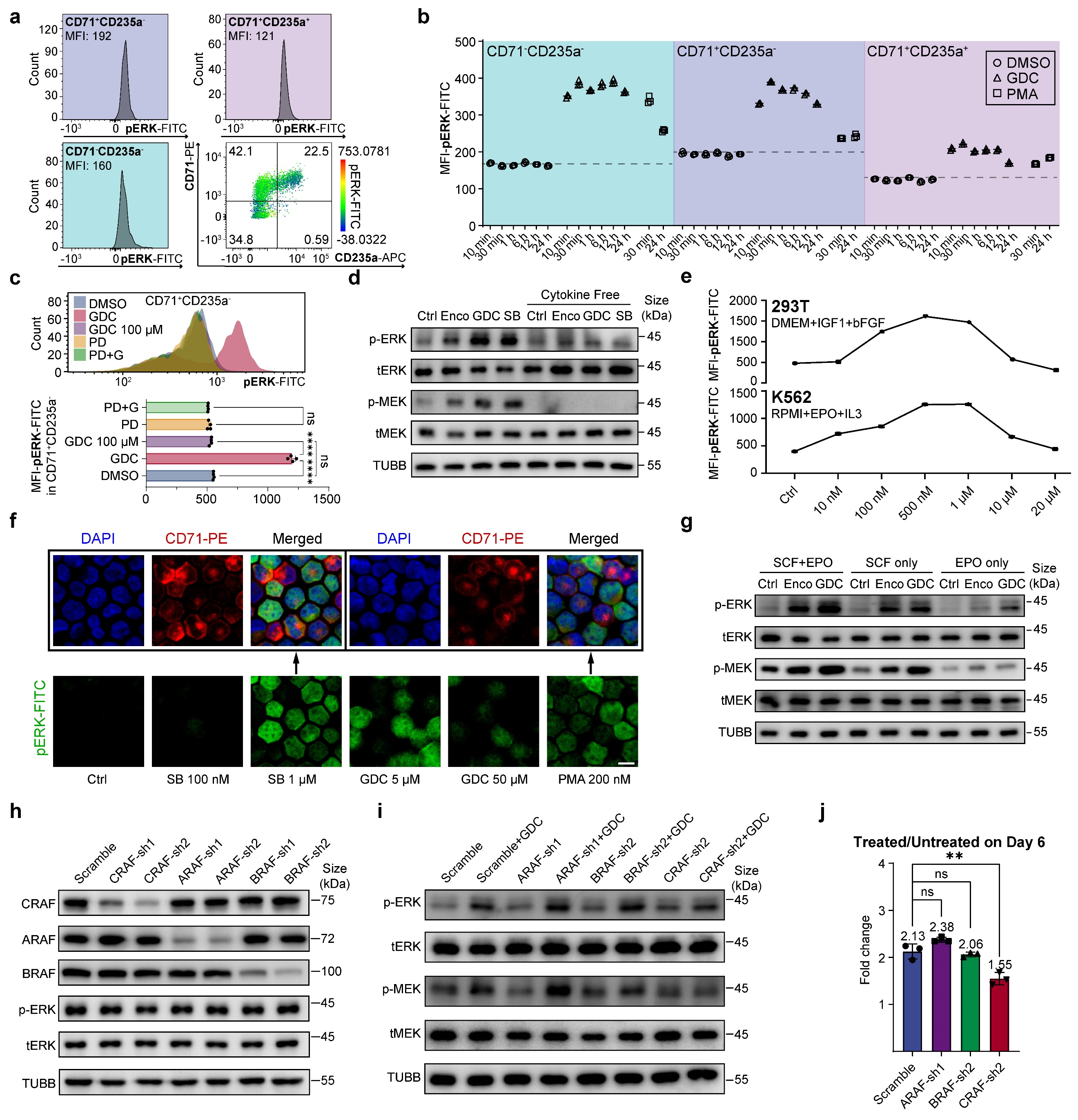
**

**Figure. S3. BRAF inhibitors-induced paradoxical MAPK activation relied on downstream MEK/ERK signaling, the presence of cytokines, and CRAF.**

**a** Intracellular flow cytometry analysis and pERK median fluorescence intensity (MFI) of each subpopulation cells were performed on Day 7 erythroid differentiated UCB-CD34^+^ cells. The color axis shows the fluorescence intensity of pERK-FITC in different subpopulations. **b** MFI statistics of intracellular flow cytometry of phosphorylated-ERK-FITC of 7-day differentiated erythroid cells from UCB-CD34^+^ treated with 2 μM GDC or 200 nM PMA for different time point. The dashed line indicates the fluorescence intensity of the negative control of different subpopulation. n = 3. **c** (Top) Representative histogram showing the median fluorescence intensity (MFI) of pERK in CD71^+^CD235a^-^ erythroid progenitor cells on Day 9 of erythroid differentiation in UCB-CD34^+^ cells treated with the indicated compounds for 30 min, as assessed by intracellular flow cytometry. (Bottom) Corresponding statistics of MFI-pERK in CD71^+^CD235a^-^ erythroid progenitor cells. n = 4. PD, 1 μM PD-0325901; G or GDC, GDC-0879 2 μM. The asterisks represent statistical differences obtained through one-way ANOVA test in MFI. **d** Immunoblotting analysis of MAPK/ERK signaling activation in UCB-CD34^+^ derived erythroid cells on normal differentiation Day 9, under control conditions or treated with Encorafenib (0.5 μM), GDC-0879 (2 μM), or SB-590885 (0.5 μM), with or without cytokines for 30 min. “Cytokine free” indicates the absence of SCF, EPO and IL3. **e** MFI analysis of intracellular pERK-FITC in 293T cells and K562 cells after 30 min treatment of different concentrations of SB in the presence of cytokines. For 293T: 50 ng/mL IGF1 and 50 ng/mL bFGF; K562: 10 IU/mL EPO and 20 ng/mL IL3. **f** Immunofluorescence images of K562 cells supplemented with cytokines (as panel **e**) treated with different molecules for 30 min. pERK-FITC, erythroid markers CD71-PE, and DAPI. Scale bar = 10 μm. **g** Immunoblotting analysis of MAPK/ERK signaling activation in UCB-CD34^+^ derived erythroid cells on differentiation Day 6, in the presence or absence of BRAF inhibitor treatment or cytokines (3 IU/mL EPO, 50 ng/mL SCF) for 30 min. **h** Immunoblotting to assess RAF monomer knockdown efficiency through lentivirus-mediated shRNA in UCB-CD34^+^ derived erythroid cells on differentiation Day 9. **i** Immunoblotting analysis of MAPK/ERK signaling activation in UCB-CD34^+^ derived erythroid cells with or without RAF monomer knockdown by lentivirus-mediated shRNA on normal differentiation Day 9, in the presence or absence of BRAF inhibitor treatment for 30 min. **j** Fold change in cell proliferation following 6 days of treatment with 2 μM GDC-0879 in GFP^+^-sorted Day 1 UCB-CD34^+^ derived erythroid cells with different RAF monomer knockdowns in erythroid differentiation culture. GFP^+^ cells indicate successful lentiviral transduction. Data are shown as the mean ± SD. A one-way ANOVA test was used for the comparison in panel **c** and **j**. ns, not significant; **, *P* < 0.01, ****, *P* < 0.0001.

Figure. S4.


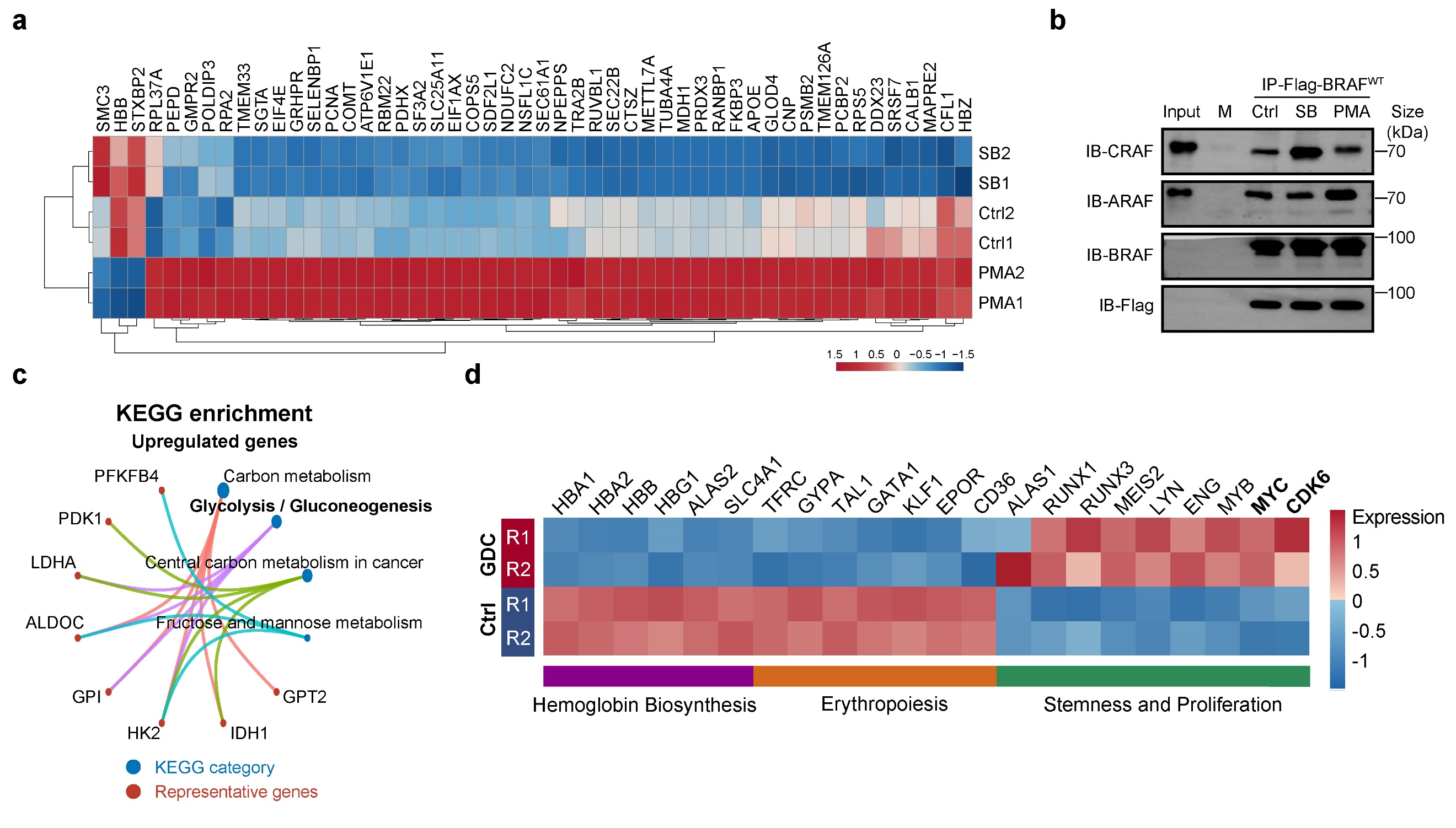


**Figure. S4. BRAF inhibitors mainly downregulated the interacting proteome of BRAF in the proteome and showed delayed erythroid development in the transcriptome.**

**a** Heatmap of the top 50 most significant changed proteins interacting with the 3×Flag-BRAF under different drug treatment conditions in K562 cells (the top 50 lowest *p*-values after multiple testing correction using the Benjamini-Hochberg method). **b** Immunoprecipitation (IP) of Flag-tagged BRAF^WT^ using an anti-flag antibody and followed by immunoblotting (IB) for CRAF, ARAF, BRAF, and Flag in different experimental conditions in K562 cells (Chemicals treated for 30 min). "Input" refers to the total lysate before immunoprecipitation, "M" denotes the molecular weight marker, and "Ctrl," "SB," and "PMA" correspond to control (DMSO), 1 μM SB (SB-590885), and 200 nM PMA treatments, respectively. **c** Representative KEGG enrichment analysis of upregulated DEGs in the 2 μM GDC-treated 72h group compared to the control group. **d** Heatmap of representative gene expression levels from RNA-seq analysis of CD71^+^ erythroid progenitor cells treated with 2 μM GDC for 72 h compared to the control group.

Figure. S5.


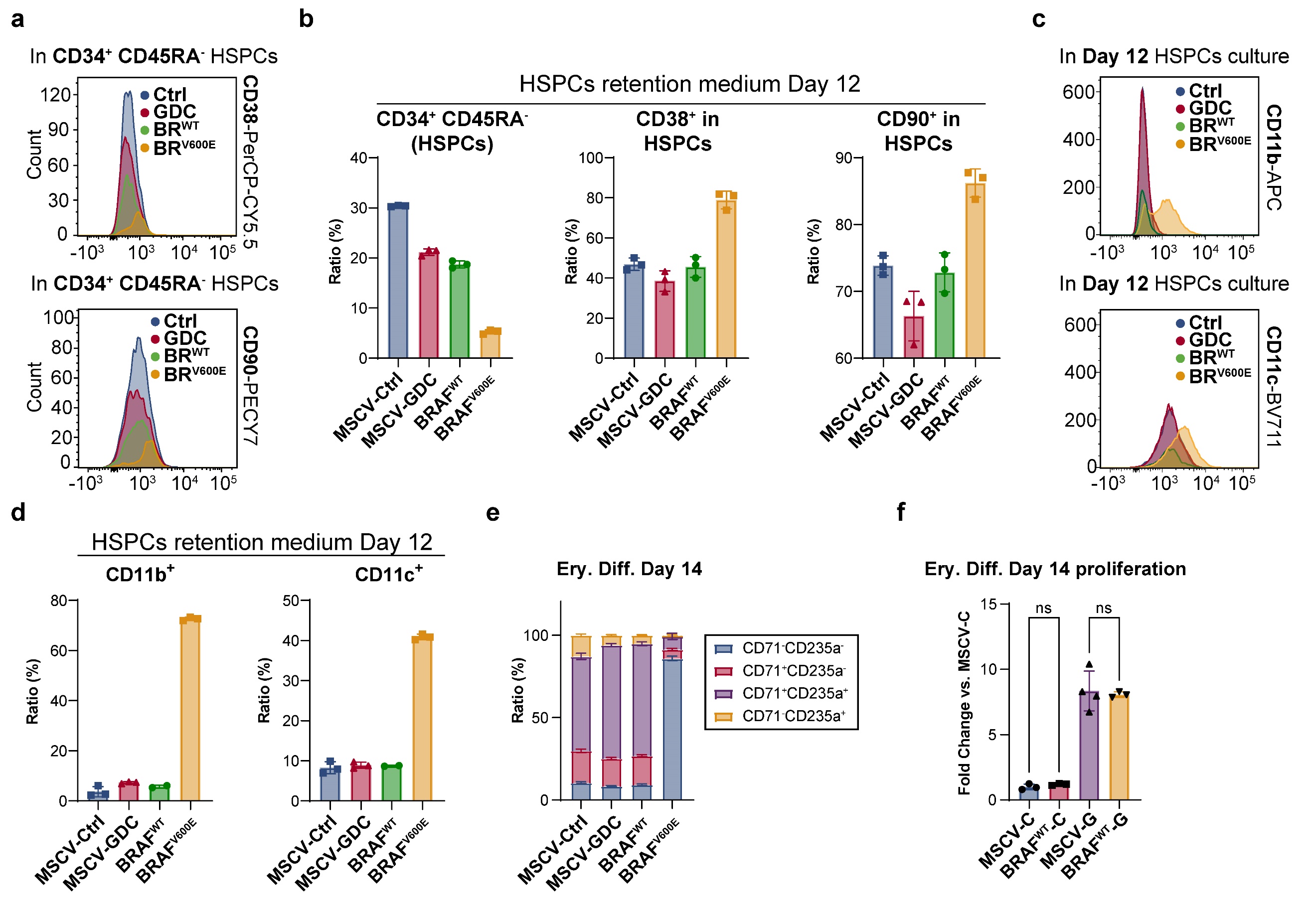


**Figure. S5. BRAF^V600E^ overexpression, neither BRAF inhibitors nor BRAF^WT^ overexpression, disrupted HSPC retention, erythroid differentiation and led to differentiation bias.**

**a** Flow cytometry analysis of surface expression levels of CD38 and CD90 in CD34^+^CD45RA^-^ UCB-HSPCs overexpressing BRAF^WT^ or BRAF^V600E^, or MSCV-GFP treated with 2 μM GDC or control (DMSO), in HSPCs retention medium on Day 12. **b** Quantification of different HSPC subpopulations on Day 12 in HSPC retention medium. CD34^+^CD45RA^-^ are considered hematopoietic stem and progenitor cells (HSPCs). **c** Flow cytometry analysis of surface expression levels of CD11b and CD11c in UCB-CD34^+^ cells overexpressing BRAF^WT^ or BRAF^V600E^, or MSCV-GFP treated with 2 μM GDC or control (DMSO), in HSPCs retention medium on Day 12. CD11b is a marker for monocytes/macrophages, and CD11c is a marker for dendritic cells; both are highly expressed on Langerhans cells. **d** Quantification of cell ratio with myeloid cell marker CD11b and CD11c in HSPCs at Day 12 in HSPC retention medium. **e** Quantification of erythroid populations (CD71 and CD235a expression) at Day 14 of differentiation in UCB-CD34^+^ cells overexpressing BRAF^WT^ or BRAF^V600E^, or MSCV-GFP treated with 2 μM GDC or control (DMSO). **f** Cell proliferation fold change of different groups compared with MSCV-GFP control group (DMSO) on differentiation Day 14 (treated for 9 days). n = 3 or 4. Data are shown as the mean ± SD. An unpaired Student’s *t*-test was used for the comparison between two groups. ns, not significant.

Figure. S6.


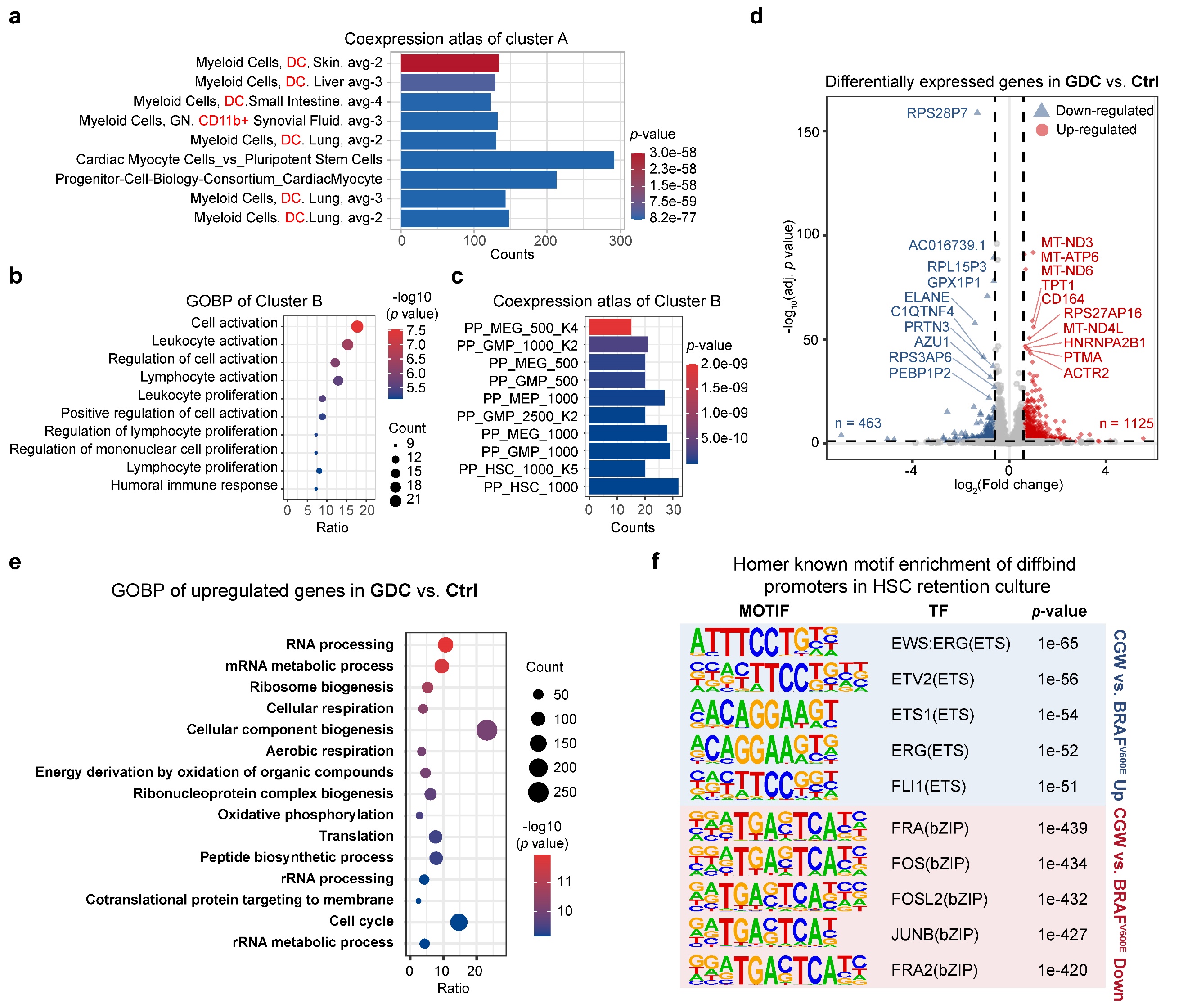


**Figure. S6. BRAF^V600E^ showed a distinctive transcriptome and epigenome landscape compared to the other groups.**

**a** Enrichment results from the Coexpression atlas for gene cluster A in **Fig. 5a** of UCB-CD34^+^ cells on Day 6 of treatment/transduction in HSPCs retention medium. DC, dendritic cells. **b** Enrichment results from the GOBP for gene cluster B in **Fig. 5a** of UCB-CD34^+^ cells on Day 6 of treatment/transduction in HSPCs retention medium. **c** Enrichment results from the Coexpression atlas for gene cluster B in **Fig. 5a** of UCB-CD34^+^ cells on Day 6 of treatment/transduction in HSPCs retention medium. **d** Volcano plot of the differentially expressed genes (DEGs) between GDC-treated and control groups of UCB-CD34^+^ cells on Day 6 of treatment/transduction. DEGs were identified with a cutoff fold change > 1.5 and FDR < 0.1. The top 10 up- and down-regulated DEGs ranked by lowest *p*-value are shown. **e** Gene Ontology biology process (GOBP) analysis of upregulated DEGs in panel **d**. **f** Known motif enrichment analysis of differential ATAC-seq peaks in promoter regions from the Ctrl, GDC and BRAF^WT^ (CGW) group’s common region and the BRAF^V600E^ group, in the HSPC retention system on Day 6 using HOMER software. The table displays transcription-factor-binding motifs enriched in promoters in the ATAC-seq data of the CGW group (Top, blue shade) and the BRAF^V600E^ group (Bottom, red shade). The Ctrl and GDC groups were transduced with the MSCV-GFP empty vector and treated with DMSO or 2 μM GDC-0879, respectively.

Figure. S7.


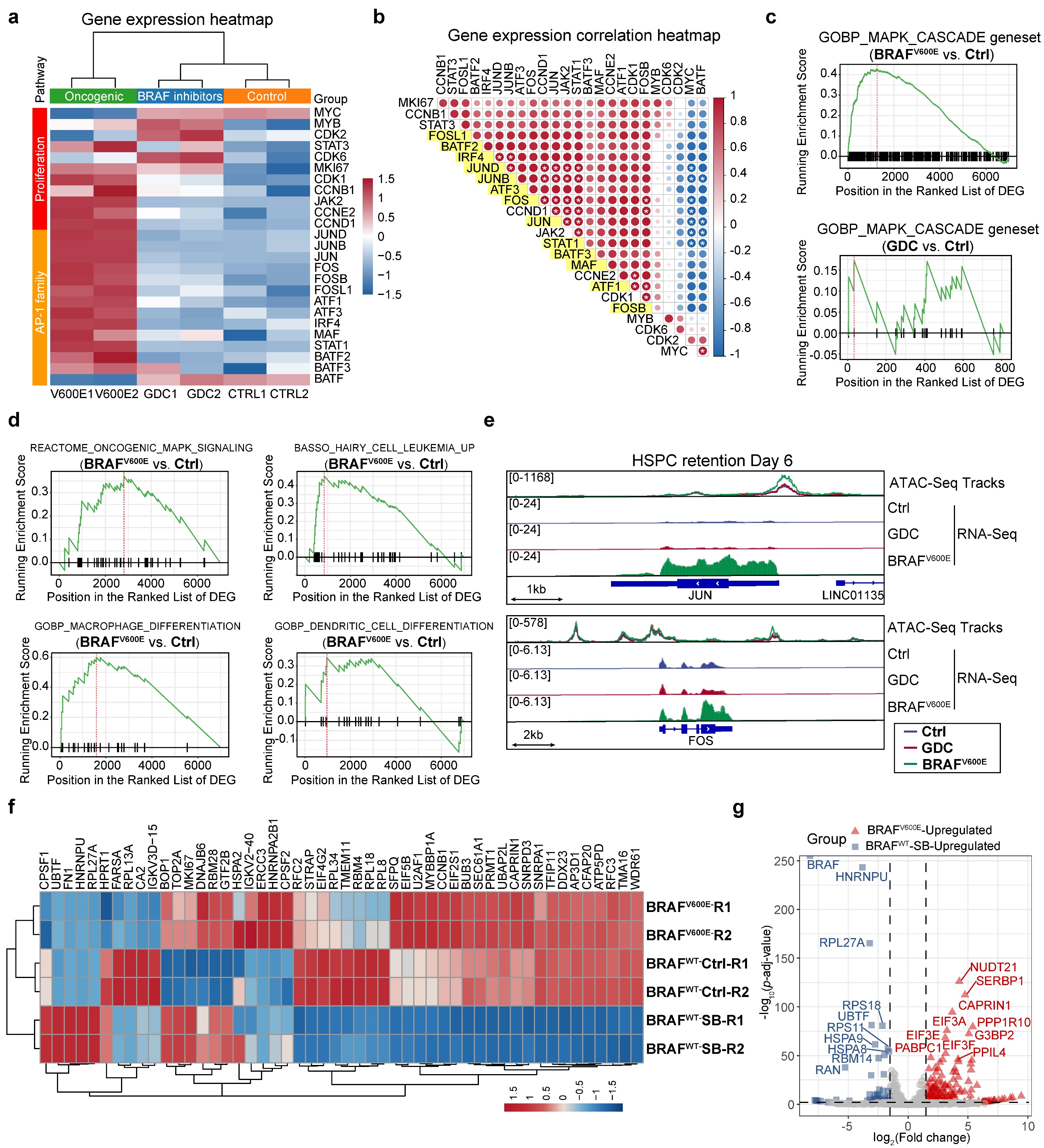


**Figure. S7. BRAF^V600E^ drove the distinctive multi-omics phenotype via hyperactivation of the AP-1 family.**

**a** Heatmap showing representative proliferation and AP-1 family gene expression levels from RNA-seq of UCB-CD34^+^ cells on Day 6 of treatment/transduction in HSPCs retention medium. **b** Coexpression correlation map of all genes in panel **a.** The genes in panel **a** are listed along the diagonal, and the Coexpression correlation between each gene and others is represented by colored dots in a row next to the respective gene. AP-1 family genes are highlighted in yellow. Asterisks indicate genes with Coexpression coefficients having a *p*-value < 0.001. **c** Gene Set Enrichment Analysis (GSEA) analysis of GOBP_MAPK_CASCADE in BRAF^V600E^ or GDC vs. control (DMSO) group of UCB-CD34^+^ cells on Day 6 of treatment/transduction in HSPCs retention medium. **d** Gene Set Enrichment Analysis (GSEA) of the shown pathways comparing BRAF^V600E^ with the control group in UCB-CD34^+^ cells on Day 6 of treatment/transduction in HSPCs retention medium. **e** Peaks of RNA-seq and ATAC-seq at the *JUN* and *FOS* loci in UCB-CD34^+^ cells on Day 6 of treatment/transduction in HSPCs retention medium. **f** Heatmap of the top 50 most significantly altered interacting proteins with the 3×Flag-BRAF^WT^ or BRAF^V600E^ under different drug treatment conditions in 293T cells (the top 50 lowest *p*-value after multiple testing correction using the Benjamini-Hochberg method). **g** Volcano plot of 3×Flag-BRAF^WT^ or BRAF^V600E^ interacting proteins in IP-MS of SB-treated BRAF^WT^ overexpressed and BRAF^V600E^ overexpressed groups in 293T cells. cutoff: *p-adj* < 0.01, foldchange > 2. The Ctrl and GDC groups were transfected with the MSCV-GFP empty vector and treated with DMSO or 2 μM GDC-0879, respectively.

Figure. S8.

**
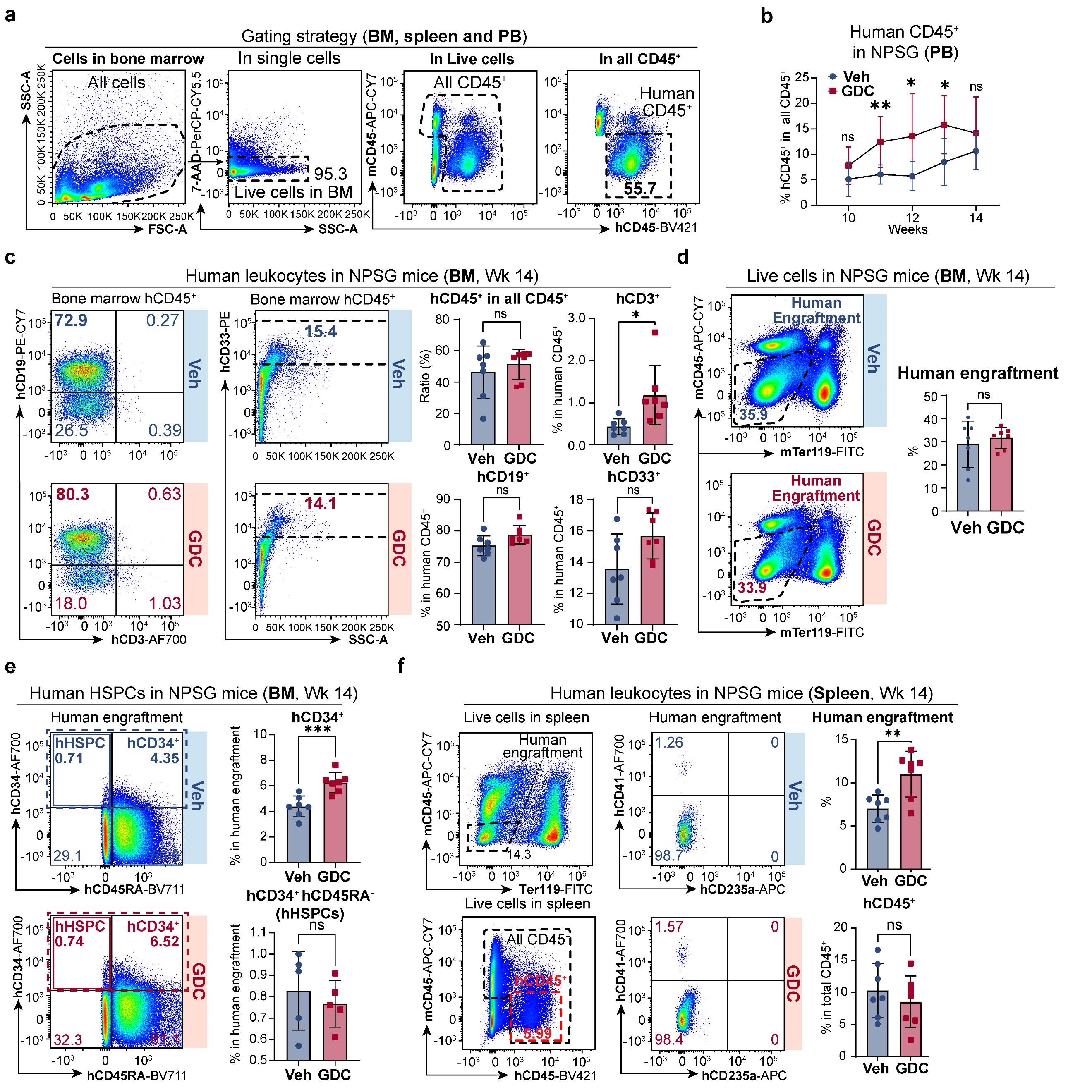
**

**Figure. S8.** **BRAF inhibitors promoted human erythropoiesis and hematopoiesis in NPSG mouse model****.**

**a** General gating strategy to identify live cells, total CD45^+^ cells and human CD45^+^ cells in bone marrow, spleen, and peripheral blood samples of NPSG and NCG-X mice. **b** Statistics on the proportion of human leukocytes (hCD45^+^) in peripheral blood of NPSG mice during GDC treatment from Week 10 to Week 14. **c** (Left) Representative gating strategy of flow cytometry identifying human chimeric leukocytes and its compositions of human leukocytes proportions in hCD45^+^ on Week 14 in NPSG mice bone marrow. CD3 is a marker for T lymphocytes; CD19 is a marker for B lymphocytes; CD33 is a marker for myeloid cells. (Right) Statistics of human leukocytes ratio and its compositions in NPSG mice bone marrow on Week 14. **d** (Left) Representative gating strategy of flow cytometry identifying human engraftment ratio on Week 14 in NPSG mice bone marrow, and (Right) corresponding statistics of human engraftment ratio in NPSG mice BM. **e** (Left) Representative gating strategy of flow cytometry identifying human HSPCs and (Right) corresponding statistics on Week 14 in NPSG mice bone marrow. **f** (Left and middle) Gating strategy of flow cytometry to identify the ratio of human leukocytes to total leukocytes and human engraftment in the spleen and (Right) corresponding statistics in Week 14 in NPSG mice spleens. Error bars represent the mean ± SD. An unpaired two-tailed Student's *t*-test was performed for the statistical comparison between two groups (ns, *P*＞0.05; *, *P* < 0.05; **, *P* < 0.01; ***, *P* < 0.001). n = 7 in each group in NPSG mice model. Each dot represents one mouse.

Figure. S9.


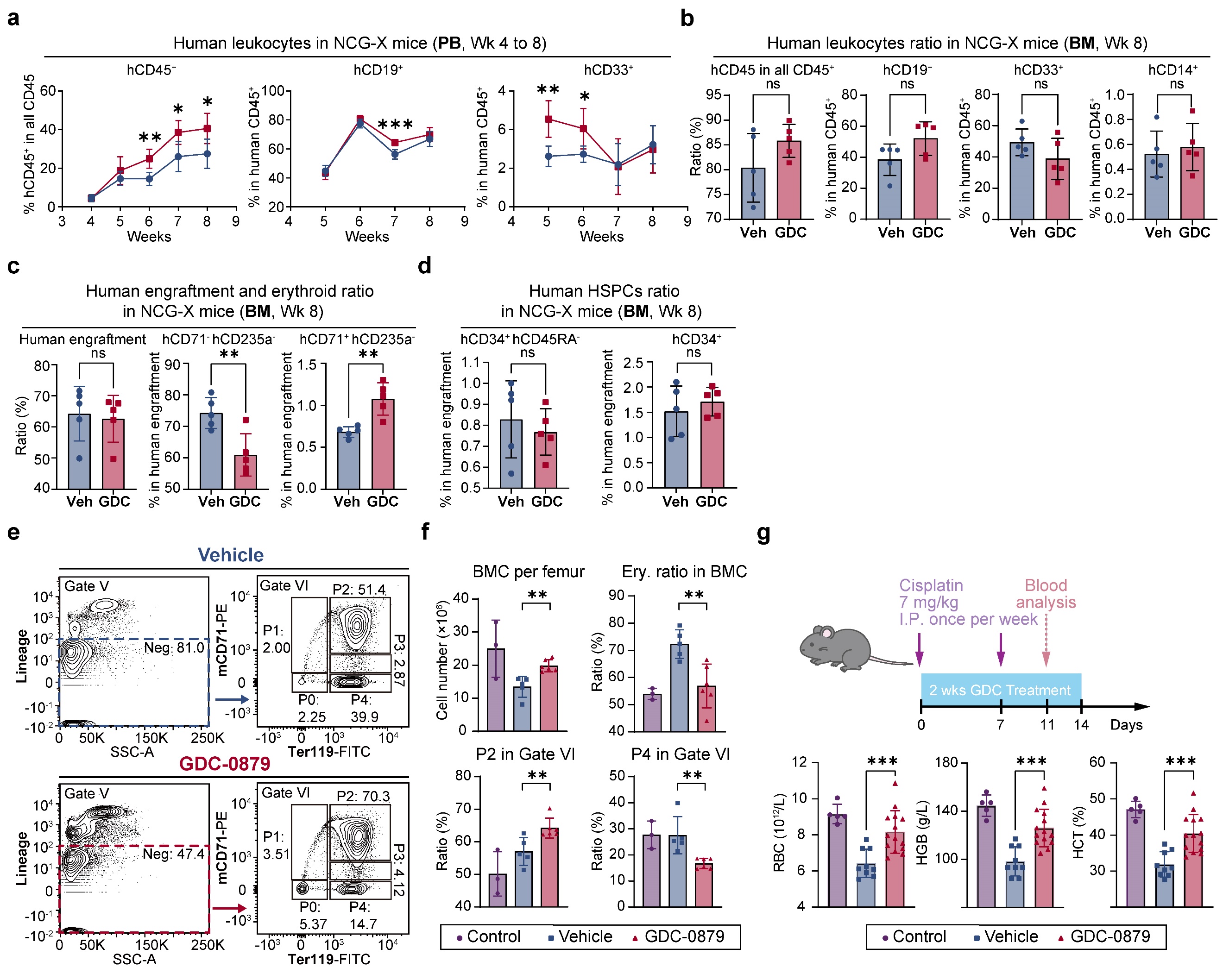


**Figure. S9. BRAF inhibitors promoted human erythropoiesis and hematopoiesis in NCG-X mouse model and mouse erythropoiesis in cisplatin-induced myelosuppression model.**

**a** Statistics on the proportion of human leukocytes (hCD45^+^) and the leukocyte ratio in the peripheral blood of NCG-X mice during GDC treatment from Week 4 to 8. **b** Statistics of human leukocyte ratio and its compositions in NCG-X mice bone marrow at Week 8. **c** Human engraftment and erythroid cell ratio statistics in NCG-X mice bone marrow at Week 8. **d** Human HSPCs statistics in NCG-X mice bone marrow at Week 8. n=5 in each group in NCG-X mouse model. Each dot represents one mouse. **e** Representative flow cytometric analysis of erythroid lineage in bone marrow live cells (BMC) from randomly selected mice (n=3 to 6) from each group in cisplatin-induced myelosuppression model in **Fig. 6g** at Week 3. Lineage positive cells in Gate V consist of B220^+^, CD3^+^, GR1^+^ or CD11b^+^ cells. **f** Quantification of BMC and the erythroid population ratio in panel **e**. Erythroid ratio defined as: CD71^+^ or TER119^+^ cells in BMC. n=3~6 in each group randomly selected from this cisplatin-induced myelosuppression model (**Fig. 6g**). **g** Schematic representation of another cisplatin-induced anemia model (protective effect) and blood cell parameter measured on Day 11. The timeline for cisplatin and vehicle/GDC treatment differs from **Fig. 6g**. In this model, control group, n = 5; vehicle group, n = 9; GDC group, n=14. Each dot represents one mouse. Error bars represent the mean ± SD. A two-tailed unpaired Student's *t*-test was performed for the statistical comparison between two groups (ns, *P*＞0.05; *, *P* < 0.05; **, *P* < 0.01; ***, *P* < 0.001).

**Table S1. Antibodies for flow cytometry**

| **Name** | **Clone** | **Conj.** | **Company** | **Cat. No.** |
| --- | --- | --- | --- | --- |
| hCD34 | 4H11 | PE | Invitrogen | A16203 |
| hCD34 | 4H11 | PerCP | Invitrogen | 46-0349-41 |
| hCD117 | 104D2 | PE | Invitrogen | CD11704 |
| hCD71 | OKT9 | PE | Invitrogen | 12-0719-42 |
| hCD71 | OKT9 | FITC | Invitrogen | 11-0719-42 |
| hCD235a | HIR2 | PE | Invitrogen | MA1-19661 |
| hCD235a | HIR2 | APC | Invitrogen | 17-9987-42 |
| hCD11b | ICRF44 | PE-Cy5 | Invitrogen | 555389 |
| Human Hematopoietic Lineage | RPA-2.10, OKT3, 61D3, C、B16, HIB19, TULY56, HIR2 | APC | Invitrogen | 22-7776-72 |
| hCD10 | HI10a | APC | BioLegend | 312210 |
| hCD90 | 5E10 | APC | BD | 559869 |
| hCD45RA |  | APC | BD | 550855 |
| hCD7 |  | APC | BD | 561604 |
| hCD123 |  | APC | BD | 560087 |
| hCD135 | BV10A4H2 | APC | BioLegend | 313308 |
| hCD41 |  | APC | BD | 559777 |
| Annexin V |  | FITC | BD Pharmingen | 556547 |
| DAPI |  |  | Invitrogen | D3571 |
| Propidium iodide |  |  | Invitrogen | P1304MP |
| Hoechst 33342 |  |  | Invitrogen | H3570 |
| 7-AAD |  |  | Invitrogen | 00-6993-50 |
| mCD117 | 2B8 | FITC | BioLegend | 105806 |
| Streptavidin |  | BV711 | BioLegend | 405241 |
| mCD71 | RI7217 | PE | BioLegend | 113808 |
| mTER119 | TER-119 | FITC | Invitrogen | 2056566 |
| mLineage |  | PerCP-Cy 5.5 | BD | 51-9006964 |
| mGR1 |  | Biotin | BD | 51-01212J |
| mCD11b |  | Biotin | BD | 51-01712J |
| mCD3e |  | Biotin | BD | 51-01082J |
| mCD45R |  | Biotin | BD | 51-01122J |
| mCD45 | 30-F11 | APC-Cy7 | BD | 557659 |
| hCD45 | H130 | BV421 | BD | 563879 |
| hCD19 | SJ25C1 | PE-Cy7 | Invitrogen | 25-0198-41 |
| hCD3 | UCHT1 | AF700 | Invitrogen | 56-0038-80 |
| hCD33 | HIM3-4 | PE | Invitrogen | 12-0339-42 |
| hCD14 | 6ID3 | FITC | Invitrogen | 11-0149-41 |
| hCD11b | ICRF44 | APC | Invitrogen | 17-0118-41 |
| hCD11c |  | BV711 | BD | 563130 |
| hCD41 | MEM-06 | AF700 | Invitrogen | MA5-28562 |
| hCD45RA | HI100 | BV711 | BD | 563733 |
| hCD38 | HB7 | BV605 | BD | 562665 |
| hCD90 | 5E10 | PE-Cy7 | BD | 561558 |
| hCD34 | 581 | AF700 | BD | 561440 |
| Goat anti-Rabbit IgG F(ab')2 Secondary Antibody |  | FITC | Invitrogen | 31573 |

**Table S2. Antibodies for immunoblotting**

| **Name** | **Company** | **Cat. No.** |
| --- | --- | --- |
| B-Raf (55C6) | Cell Signaling | 9433T |
| Phos-Mek1/2 (S217/221) | Cell Signaling | 9154S |
| beta-Tubulin (9F3) | Cell Signaling | 5346S |
| B-Raf (D9T6S) | Cell Signaling | 14814S |
| Mek1/2 (L38C12) | Cell Signaling | 4694S |
| Phos-p44/42 MAPK (T202/Y204) | Cell Signaling | 4370S |
| p44/42 MAPK(Erk1/2) (137F5) | Cell Signaling | 4695S |
| c-Raf (D4B3J) | Cell Signaling | 53745S |
| A-Raf (D2P9P) | Cell Signaling | 75804S |
| Phos-c-Raf (S338) | Cell Signaling | 9427S |
| Phos-c-Raf-S338 | ABclonal | AP0498 |

**Table S3. shRNA for knockdown**

| **Name** | **Sequence (5’ to 3’)** |
| --- | --- |
| Scramble | CAGGAATTATAATGCTTATCTA |
| ARAF-sh1 | TTTCGTCCCTTGATGAGTCGGT |
| ARAF-sh2 | TAGATTTTGTCAAGATGGGCTG |
| BRAF-sh1 | TTAAATTCTACTGACTTCCTAA |
| BRAF-sh2 | TTCTGTACTACAACGCTGGTGA |
| CRAF-sh1 | TAGGAGTAGACATCCGACTGGA |
| CRAF-sh2 | TTGTCTTAGAAGGATCTGTGAG |

**Table S4. Primers for qRT-PCR**

| **Name** | **Sequence (5’ to 3’)** |
| --- | --- |
| m*Gapdh*-F | AGAGTGTTTCCTCGTCCCGT |
| m*Gapdh*-R | GAGGTCAATGAAGGGGTCGT |
| m*Rpl11*-F1 | CCTCAATATCTGCGTCGGGG |
| m*Rpl11*-R1 | TTCTCCGGATGCCAAAGGAC |
| m*Rpl11*-F2 | GGAAATTCTGGAGAAAGGCCTGA |
| m*Rpl11*-R2 | AAGTCCAGGCCGTAGATCCC |
